# Overview on magnetically recyclable ferrite nanoparticles: synthesis and their applications in coupling and multicomponent reactions

**DOI:** 10.1039/d1ra03874e

**Published:** 2021-09-01

**Authors:** Runjhun Tandon, Nitin Tandon, Shripad M. Patil

**Affiliations:** Department of Chemistry, School of Chemical Engineering and Physical Sciences, Lovely Professional University Phagwara-144411 India tandonnitin12004@gmail.com patilshripad55@gmail.com

## Abstract

Nanocatalysis is an emerging area of research that has attracted much attention over the past few years. It provides the advantages of both homogeneous as well as heterogeneous catalysis in terms of activity, selectivity, efficiency and reusability. Magnetically recoverable nanocatalysts provide a larger surface area for the chemical transformations where the organic groups can be anchored and lead to decrease in the reaction time, increase in the reaction output and improve the atom economy of the chemical reactions. Moreover, magnetic nanocatalysts provide a greener approach towards the chemical transformations and are easily recoverable by the aid of an external magnet for their reusability. This review aims to give an insight into the important work done in the field of magnetically recoverable nanocatalysts and their applications in carbon–carbon and carbon–heteroatom bond formation.

## Abstract 2

Applications of ferrite nanoparticles in various chemical transformations.

## Graphical abstract

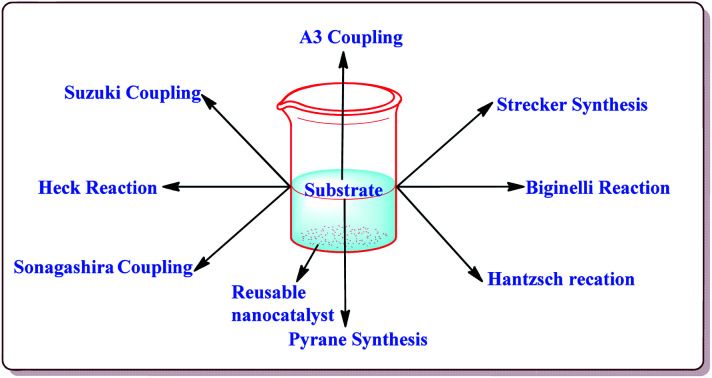

## 1. Introduction

From the recent past, catalysis has emerged as a mature area of research supported by well explained theories and explanations. It is one of the twelve principles of green chemistry. Researchers are working on the development of new catalysts which either have advantages over the existing catalytic systems or can be used for the improvement of the emerging processes in terms of cost effectiveness and ease of reactions. Design of a catalyst having desired catalytic properties for a specific chemical transformation is an art that requires the knowledge of optical, electronic, energetic and photonic efficiencies.^1–3^ Further, development of highly functionalized catalysts requires high-throughput technology.^4,5^ From the past few years, many fruitful efforts have been made in the area nanocatalysts that provide advantages of homogeneous as well as of heterogeneous catalysis in terms of activity, selectivity, efficiency and reusability.^6–8^ This can be attributed to the nanostructure, quantum size and electronic effects of the nanoparticles (NPs).^9–11^

### 1.1. Nano-catalysis as a tool for green and sustainable chemistry

Green and sustainable chemistry is one of the key research areas which can pave a way to meet the continuously increasing demand of the population which is expected to be 9 billion by the year of 2050. Nano-catalysis is essential for sustainable and green chemistry in terms of use of green reagents which avoid the use of harmful and toxic chemicals, using solvent free reactions conditions which reduce the effluent treatment load, moderate reaction conditions, less reaction time and reusability of the catalysts. These characteristics of the nanocatalysts have led to their numerous applications in various organic transformations.^12–17^ Further, these nanocatalysts provide simple and eco-friendly methods for synthetic transformations with excellent yields and selectivity.^18–23^ Many attempts have been made for the synthesis of nanoparticles that can participate in green chemistry and can be reused for many cycles without appreciable loss in their catalytic activity.^24–27^ Heterogeneous nano-catalysts like zeolites, metal oxides, clay particles *etc.* have been widely used in the industrial sector for various organic transformations and have proven to be more effective than the homogeneous catalysts in terms of ease of work up after the completion of the reaction, fewer chances of formation of by-products and recyclability of the catalysts.^28,29^

### 1.2. Fe_3_O_4_ nanoparticle catalyst and Fe_3_O_4_@SiO_2_ magnetic catalyst

From the last few years, preparation of ferrite nanoparticles has emerged as the key research area in the field of catalysis. In 1930, Frankel *et al.* reported that the conventional ferrite nanoparticles are of the size between 10–100 nm. Below 10 nm, ferrite nanoparticles possessed super paramagnetic properties.^18^ Ferrite nanoparticles have extensively been studied for their applications in biomedical field.^30,31^ medical diagnostic,^32,33^ multimodal imaging^34,35^ and medical therapy.^36–39^ The preparation of these nanoparticles has been achieved through various methods like co-precipitation, thermal decomposition reduction technique, and sol–gel method. Further, these nanoparticles have been coated by silica and various metals like TiO_2_, Cu, Zn, Ni, Co, Cu, Pd, Pt *etc.* to explore their catalytic activities in various important organic transformations.^19–22^

### 1.3. Scope of the present review

**Fig. 1:**
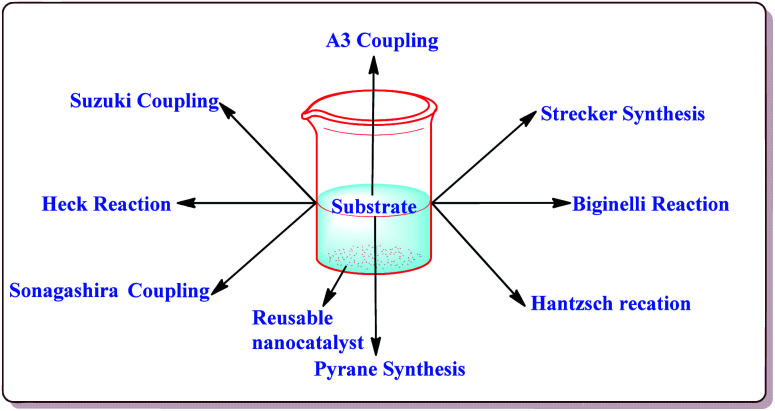
Scope of the ferrite nanocatalysts.

Silica coated ferrite nanoparticles have been well known in literature for their catalytic activity to carry out various organic transformations (Fig. 1). The main key features of these nanocatalysts include their high selectivity, excellent yields in lesser reaction time and hence these catalysts provide economical ways of synthesizing the target products with high selectivity. Further, these catalysts avoid the use of harmful chemical, reagents and solvents and thus are environment friendly. Also, due to their magnetic properties, these catalysts are easily separable after the completion of the reaction by applying external magnetic fields and can be reused for number of repeated cycles without any significant loss in their catalytic activity. Various review articles have been published from time to time on the applications of ferrite nanoparticles. Kharisov *et al.* have recently reported a mini review on the ferrite nanoparticles for the catalysis of the various processes like methanol decomposition, degradation of phenolic compounds, H_2_O_2_ decomposition, electrocatalyst for oxygen evolution reaction *etc.*,^40^ Patil *et al.* have reported a review on the synthesis of various silica coated ferrite nanoparticles.^41^ Abu-Dief *et al.* have reported the development and functionalization of magnetic nanoparticles till 2010.^42^ Gwande *et al.* have reported a review in 2013 on Fe_3_O_4_ supported catalysts for the development of sustainable methodologies.^43^ Lim *et al.* have reviewed the applicability of magnetically recyclable nanocatalysts for catalysing various organic reactions till 2010.^44^ In 2009, Polshettiwar *et al.* have reported a review on the applications of palladium based silica supported nanocatalysts for various synthetic reactions like Heck reactions, Sonogashira reactions, Suzuki–Miyaura reactions *etc.*^45^ The main scope of the present review is to compile the important research work done in the field of the magnetically recoverable ferrite nanoparticles from 2010 onwards. Further, the application of these nanocatalysts to catalyze various carbon–carbon and carbon–heteroatom bond formation reactions like Suzuki, Heck, Sonogashira, and A3 coupling reactions as well as multicomponent reactions like Strecker, Biginelli and Hantzsch reactions have been discussed in detail in the present review.

## 2. Synthesis of ferrite magnetic nanoparticles

### 2.1 Synthesis of Fe_3_O_4_ nanoparticles

**Fig. 2:**
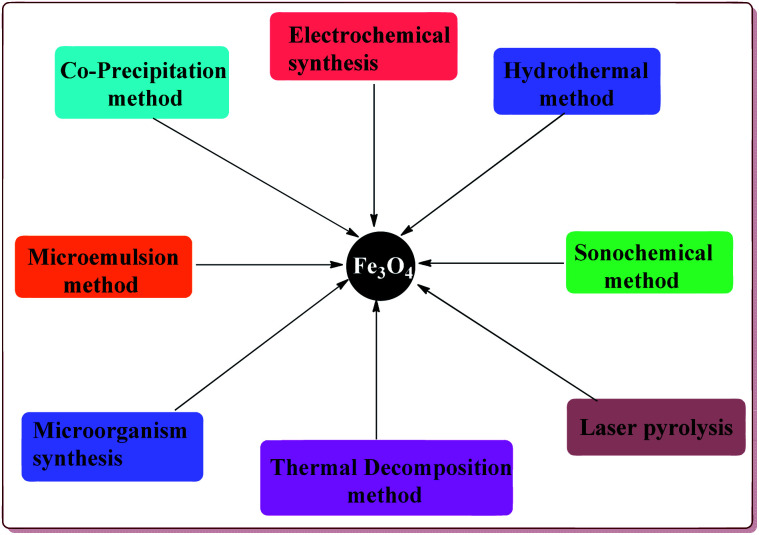
General methods for the preparation of Fe_3_O_4_ nanoparticles.

Various synthetic methods are known in literature for the preparation of Fe_3_O_4_ nanoparticles (Fig. 2). Co-precipitation is one of the common methods used for the preparation of the Fe_3_O_4_ nanoparticles which consist of mixing ferric and ferrous ions in 1 : 2 molar ratios under basic conditions at room or elevated temperature. The properties like shape and size of the nanoparticles prepared by this method depend upon the pH of the reaction mass, type of iron slats used, stirring rate and temperature during the reaction. However, this method gives Fe_3_O_4_ nanoparticles with wide particle size distribution. The synthesis of monodispersed Fe_3_O_4_ nanoparticles have also been achieved either in the absence^46^ or present of the surfactants.^47,48^ Another approach for the synthesis of monodispersed Fe_3_O_4_ nanoparticles with narrow size distribution is thermal decomposition of various salts like Fe(acac)_3_, Fe(Cup)_3_ and Fe(CO)_5_.^49–52^ In addition, microemulsion route can also be used for the synthesis of shape and size controlled Fe_3_O_4_ nanoparticles.^53–55^ Hydrothermal process is an alternate method to prepare the Fe_3_O_4_ nanoparticles with controlled size and shape that avoids the high temperature and complex reaction conditions required in the microemulsion process.^56–61^ Another versatile method used for the preparation of Fe_3_O_4_ nanoparticles is sonochemical synthesis which involve the sonication of various salts like Fe(C_2_H_3_O_2_)_2_ and Fe(acac)_3_.^62–64^ In addition to these methods, there are other reported techniques like electrochemical synthesis,^65,66^ microorganism or bacterial synthesis^67,68^ and laser pyrolysis techniques *etc.*^69^ which can also be used for the preparation of Fe_3_O_4_ nanoparticles. These ferrite nanoparticles have found their applications in various fields like biomedical,^70,71^ healthcare,^72,73^ agriculture,^74^ environmental remediation,^75^*etc.*

### 2.2. Synthesis of metal doped ferrite nanoparticle (Fe_3_O_4_@metal)

**Fig. 3:**
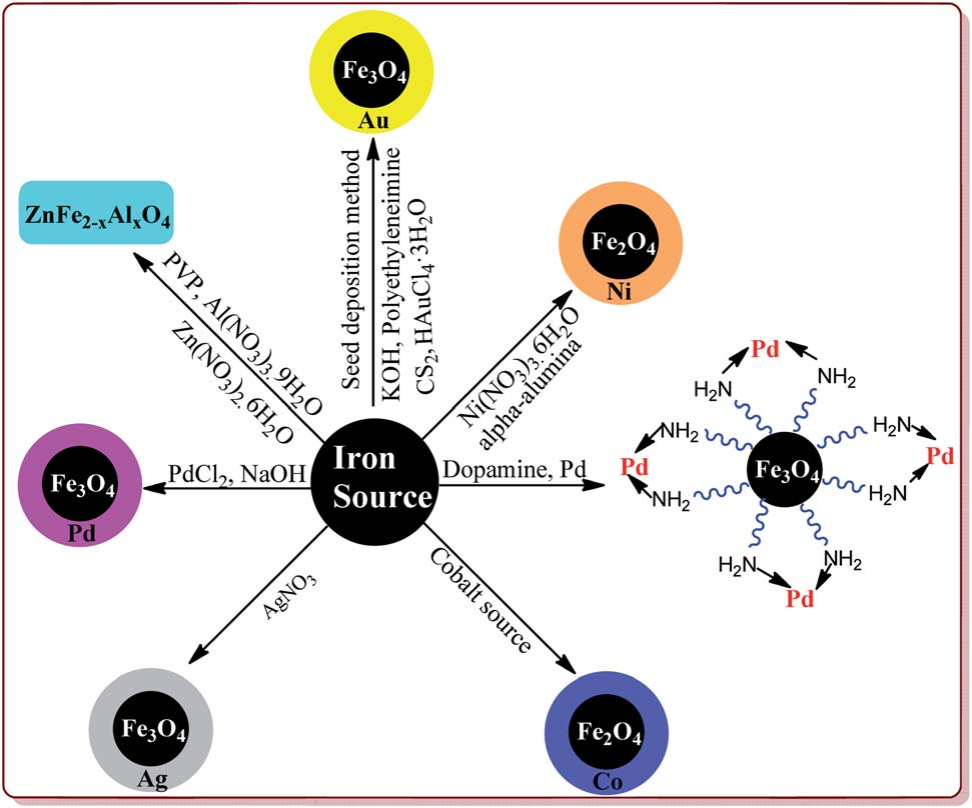
Synthesis of metal doped ferrite nanoparticle.

Metal coated ferrite nanoparticles possess different properties. For example, He *et al.* have reported the one pot synthesis of platinum-coated magnetite nanoparticles to study their magnetosensitive catalytic applications.^76^ Vaddula *et al.* have reported the synthesis of Fe_3_O_4_–Dopa–Pd nanocatalyst for catalysing Heck type reactions by reacting aqueous mixture of Fe_3_O_4_ with dopamine under sonication followed by reaction with methanolic solution of palladium.^77^ Sá *et al.* have reported the synthesis of Fe_3_O_4_ doped with Pd by impregnation method followed by chemical reduction for its application in Buchwald–Hartwig reaction.^78^ Neto *et al.* have reported an increase in the photocatalytic properties of Fe_3_O_4_ nanoparticles on its doping with Ce^4+^, Mn^2+^, CO^2+^ and Ni^2+^ metal ions by co-precipitation method.^79^ Petrov *et al.* have used thermal decomposition method to synthesize Fe_3_O_4_ nanoparticles modified with Ag by reacting Fe(NO_3_)_3_·9H_2_O and AgNO_3_ at high temperature. These prepared nanoparticles were further studied for their magnetic and magneto-optical properties.^80^ Yang *et al.* have reported the synthesis of Fe_3_O_4_/Au composites by seed deposition method for catalysing the reduction of 4-nitrophenols.^81^ Alzahrani *et al.* have reported the coating of Fe_3_O_4_ nanoparticles with Ag by using co-precipitation method from FeSO_4_·7H_2_O and AgNO_3_ and have explored its catalytic properties for photodegradation of eosin Y for the purification of industrial waste of dyes.^82^ Yang *et al.* have reported the effect of doping of magnesium on Curie temperature (*T*_c_), Magnetic properties, and heating efficiency of Zn–Co-ferrite nanoparticles.^83^ Mohamed *et al.* have reported green method for the preparation of CoFe_2_O_3_ nanoparticles as a sensor for the detection of Cu^2+^ in water samples and food products.^84^ Rather *et al.* have reported the synthesis of aluminium doped zinc ferrite nanoparticles by thermal treatment method using polyvinylpyrrolidone (PVP) as capping agent.^85^ Ishaq *et al.* have reported the synthesis of nickel ferrites nanoparticles by wet impregnation method to study their antibacterial activity (Fig. 3).^86^

### 2.3. Ionic liquid supporting ferrite nanoparticle (Fe_3_O_4_@IL)

Shikha *et al.* have reported the synthesis of manganese doped La–Ce ferrite nanoparticles by hydrothermal route using ionic liquid surfactant (ILS) by reacting La(NO_3_)_3_·6H_2_O, Fe(NO_3_)_3_·9H_2_O, cerium(iii) acetate and Mn(NO_3_)_2_·4H_2_O under sonication and observed that highly doped samples exhibited significant changes in their magnetic behaviour. Also, the transition from ferro to paramagnetism was observed for doped samples.^87^ Zhang *et al.* have achieved the *trans*-esterification of glycerol trioleate catalyzed by 1-allyl-dodecylimidazolium hydroxide ([ADIm][OH]) ionic liquids immobilized on SiO_2_/CoFe_2_O_4_ and CoFe_2_O_4_ magnetic nanoparticles. The targeted catalyst was prepared by free radical reaction between allyl groups of ionic liquid and sulfhydryl group of SiO_2_/CoFe_2_O_4_ and CoFe_2_O_4_.^88^ Dewan *et al.* have reported the use of ionic liquid stabilized magnetic cobalt nanoparticles as a catalyst to carry out aza- and thia-Michael reaction at room temperature. The catalyst was prepared by reaction of CoCl_2_·6H_2_O with NaBH_4_ in the first step followed by reaction with ionic liquid [bmim]BF_4_.^89^

### 2.4. Silica coated Fe_3_O_4_ (Fe_3_O_4_@SiO_2_) and metal coated Fe_3_O_4_@SiO_2_ (Fe_3_O_4_@SiO_2_@M)

**Fig. 4:**
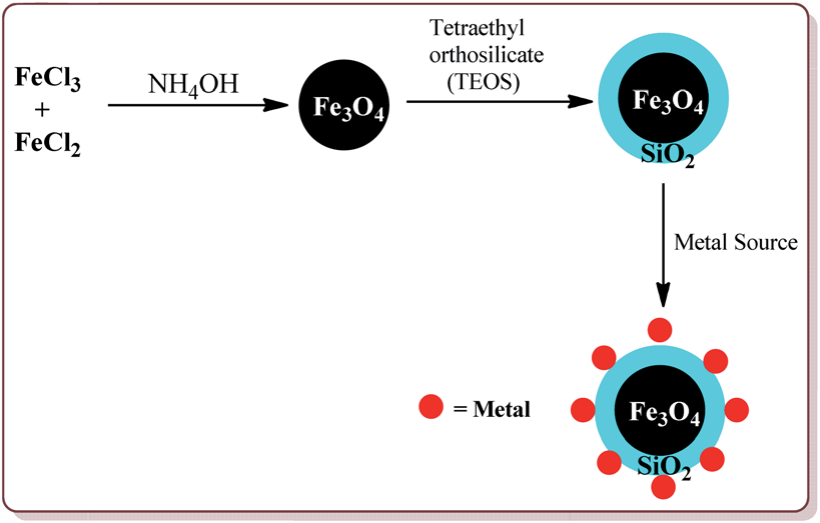
General synthesis of Fe_3_O_4_@SiO_2_ and Fe_3_O_4_@SiO_2_@M.

Many attempts have been made in literature to prepare silica and metal coated nanoparticles to study their catalytic activity for various synthetic reactions (Fig. 4). Silica as a solid support has attracted the attention of various research groups because of its wide accessibility, high porosity which facilitates the anchoring of the organic groups on its surface to generate active catalytic sites and high stability.^90^ Thangaraj *et al.* have reported the effect of the silica coating on Fe_3_O_4_ nanoparticles for lipase immobilization and further studied its application in biodiesel production. The catalyst was prepared by co-precipitation method by coating Fe_3_O_4_ nanoparticles with varying ration of SiO_2_ by using Stober method. These silica coated ferrite nanomaterials were further coated with 3-aminopropyltriethoxysilane and 3-mercaptopropyltrimethoxysilane to study their catalytic properties.^91^ Gad-Allah *et al.* have studied the role of silica content on the photocatalytic activity of TiO_2_/SiO_2_/Fe_3_O_4_ and have reported that the silica content higher than 10 wt% led to decrease in the catalytic activity. Silica and titanium coating on Fe_3_O_4_ nanoparticles was achieved by sol–gel technique.^92^ Similar types of studies have also been reported by Pang *et al.* wherein Fe_3_O_4_/SiO_2_/TiO_2_ catalyst was prepared by encapsulating Fe_3_O_4_ nanoparticles with silica followed by coating with TiO_2_ by sol–gel method. The resulted catalyst exhibited high photocatalytic properties which was evident from the photodegradation of methylene blue in UV light.^93^ The detailed synthesis and application of various silica and metal coated ferrite nanoparticles have been discussed in Section 4.

## 3. Characterization of ferrite nanoparticles

Various analytical techniques have been used in literature to characterize the magnetic nanoparticles. Surface characterization techniques are helpful to study the morphology, special distribution of the functional groups and chemical compositions.^94^ Fourier transform infrared (FT-IR) spectroscopic technique has been used in literature to confirm the type of bonding between the atoms to find out the chemical composition of the sample. Further, X-ray diffraction (XRD) study gives the information about the crystal structure and size of crystallites by using well-known Scherer formula. Transmission Electron Microscopy (TEM) technique is used to study the morphology of the crystallite structures in the sample. The magnetic properties of the ferrite nanomaterials can be studied with Vibrating Sample Magnetometer (VSM). In addition, weight loss steps in Thermal Gravimetric Analysis (TGA) studies and Inductively Coupled Plasma Atomic Emission Spectroscopy (ICP-AES) confirm the distribution of various atoms in the sample. Similar type of distribution studies can also be performed by using Scanning Electron Microscope (SEM) coupled with energy dispersive X-ray spectrometry which gives an idea about the chemical composition of the studied sample.^95–104^ Other characterization techniques include zeta potential measurement, ion-particle probe, electrophoresis, field flow fractionation, turbidimetry studies and laser microscopy *etc.*^105,106^

## 4. Applications of magnetic nanoparticles

In recent years, magnetic nanoparticles (MNPs) have attracted an increasing interest for their utilization as catalysts for the development of various green and sustainable processes with excellent yields of the products and ease of work up of reaction. These reactions have been performed under moderate reaction conditions, avoid the use of harmful chemicals and reagents and catalysts can be reused for number of cycles.^107,108^ The next part of the review is aimed to provide the application of these catalysts to catalyse various coupling, oxidation and multicomponent reactions.

### 4.1. Coupling reaction or C–C bond formation

The discovery of the homogeneous catalysis based upon transition metal elements began during the mid of 1960 and has resulted into number of important processes of industrial as well as academic importance.^109^ It has now become an important tool to form carbon–carbon and carbon–heteroatom bond formation *via* different reactions like Suzuki coupling, Sonogashira coupling, Still coupling and Heck reaction.

#### 4.1.1 Suzuki coupling reaction

**Fig. 5:**
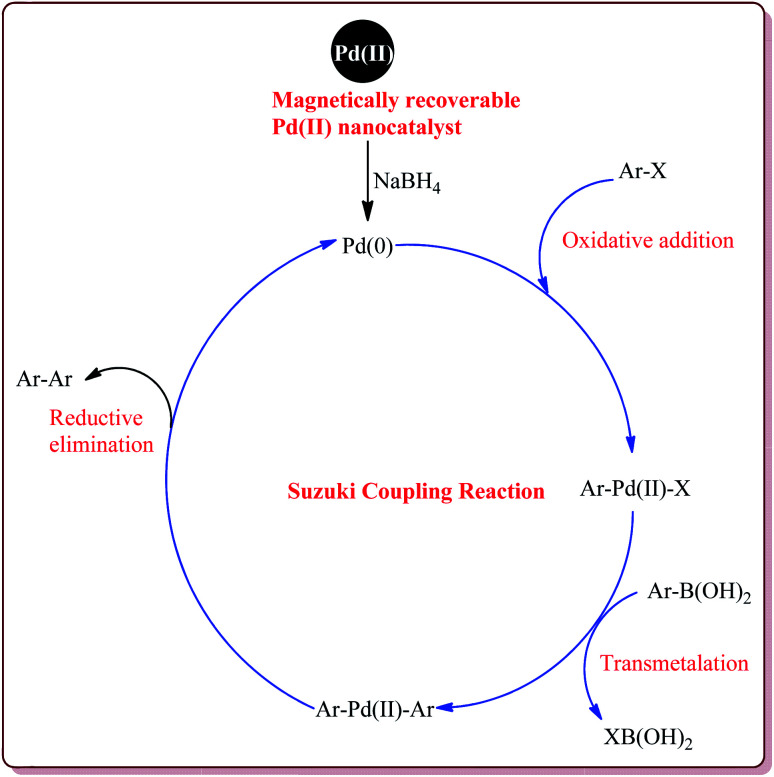
Mechanistic pathway of Suzuki coupling reaction.

The Suzuki coupling reaction, also referred as Suzuki–Miyaura reaction, is the most dominant reaction in the organic synthesis to evolve in the 20^th^ century.^110–112^ It is the most efficient method for the synthesis of the styrenes, substituted biphenyls and poly olefins and is associated with many advantages like tolerability against the presence of water and broader range of functional groups, moderate reaction conditions, easily availability of the boronic acids, high regio and stereo selectivity.^113^ Also, the boronic acids are less toxic to environment and hence are safer to use than their organostannate and organozinc derivatives making this reaction a better choice for academicians and industrialist to manufacture various targeted products. Various reviews have been published in literature discussing the applicability of this reaction for the synthesis of natural products, polymers, active pharmaceuticals and fine chemicals.^110,111,114,115^ The typical catalytic cycle of the Suzuki coupling reaction is depicted in Fig. 5 where the Palladium catalyst cycles between Pd(0) and Pd(ii) oxidation state during the catalytic cycle.

**Scheme 1:**
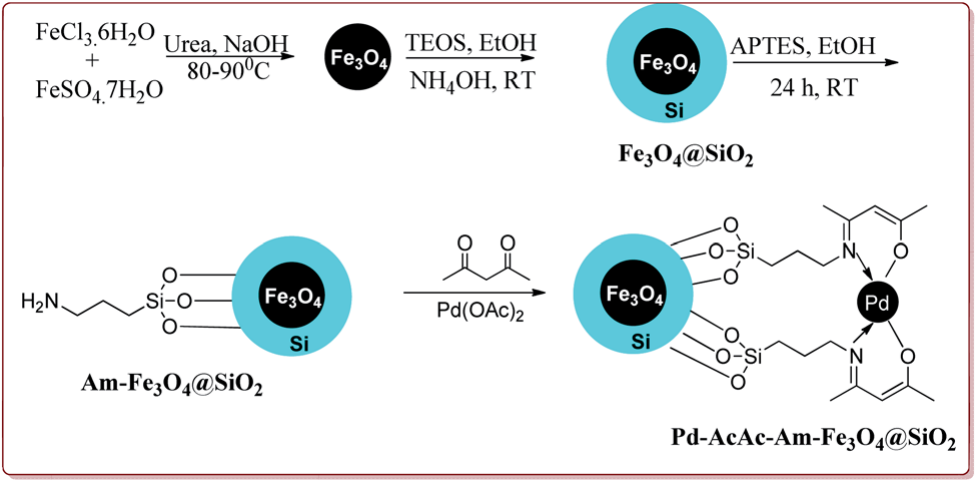
Synthetic route for the preparation of Pd-AcAc-Am-Fe_3_O_4_@SiO_2_ nanocatalyst.

**Scheme 2:**
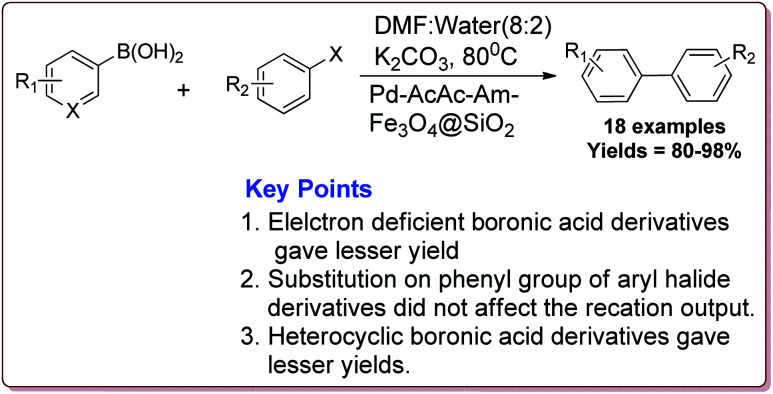
Synthesis of diarylation product of Suzuki–Miyaura cross-coupling reaction catalyzed by Pd-AcAc-Am-Fe_3_O_4_@SiO_2_.

Many transition metal base homogeneous Schiff base catalysts have been developed in the literature to carry out the Suzuki–Miyaura cross-coupling reactions which gave excellent yields as compared to heterogeneous catalysts but suffered from the disadvantage of separation of catalyst from the reaction mass which prohibit catalyst recyclability.^116–119^ Vibhute *et al.* have reported the synthesis of ferrite supported silica coated amine functionalized Schiff base–palladium(ii) nanocatalyst (Pd-AcAc-Am-Fe_3_O_4_@SiO_2_) for catalysing Suzuki–Miyaura cross-coupling reaction between aryl halides and aryl or heteroaryl boronic acid derivatives which gave excellent yield in lesser reaction time under moderate reaction conditions. The catalyst was prepared by reacting FeCl_3_·6H_2_O and FeSO_4_·7H_2_O under basic conditions followed by reaction with tetraethyl orthosilicate (TEOS) to give Fe_3_O_4_@SiO_2_. Further reaction with 3-aminopropyltriethoxysilane (APTES) and acetylacetone in the presence of palladium acetate gave target catalyst (Scheme 1). Only 30 mg of the catalyst was sufficient to catalyse the reaction. Further, the electron deficient aryl boronic acid took longer reaction time and gave lesser yields whereas heterocyclic boronic acid derivatives gave lesser yields. However, the nature of substitution on phenyl group of aryl halide did not affect the reaction output. Interestingly, the catalyst was recycled for 6 cycles and was found to be active without any significant loss in its catalytic activity (Scheme 2).^120^

**Scheme 3:**
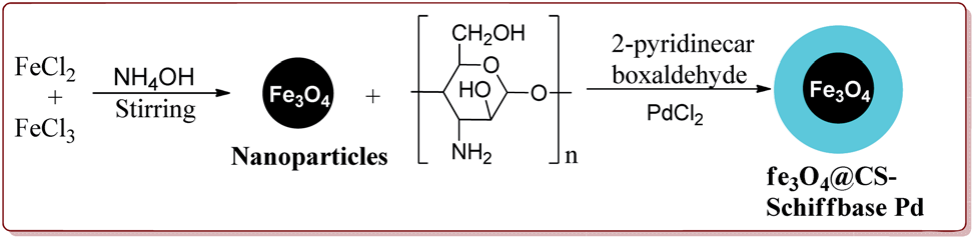
Synthesis of Fe_3_O_4_CS-Schiff base-Pd nanocatalyst.

Naghipour *et al.* have reported the synthesis of Fe_3_O_4_@chitosan-Schiff base supported Pd nanocatalyst by using chemical co-precipitation method for carbon–carbon bond formation in Suzuki–Miyaura and Heck–Mizoroki reactions (Scheme 3).

**Scheme 4:**
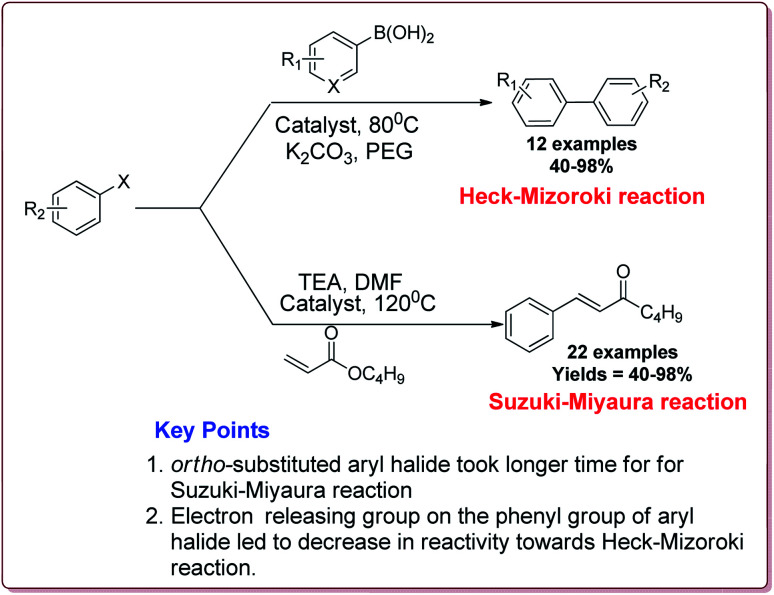
Suzuki–Miyaura and Heck–Mizoroki reactions catalyzed by Fe_3_O_4_CS-Schiff base-Pd nanocatalyst.

In general, the prepared catalyst gave excellent yields for Suzuki–Miyaura reaction irrespective of the substitution on the phenyl group of aryl halide. However, the *ortho*-substituted aryl halide derivatives took longer reaction time due to steric hindrance. On the other hand, in case of Heck–Mizoroki reaction, the presence of electron releasing group on the phenyl group of aryl halide led to decrease in the reactivity (Scheme 4). Further, 10 mg of the catalyst was sufficient to catalyse the reactions and the catalyst was used 5 times without change in its catalytic activity to much extent.^121^

**Scheme 5:**
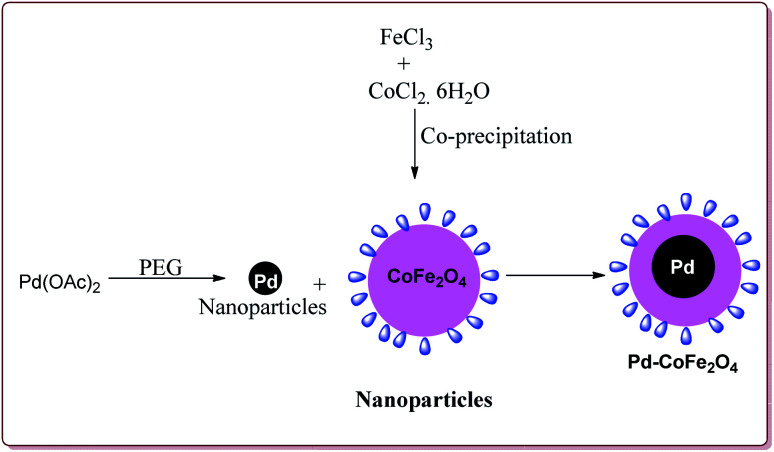
Synthesis of Pd–CoFe_2_O_4_ magnetic nanocatalyst.

Senapati *et al.* have reported the synthesis of Pd–CoFe_2_O_4_ nanocatalyst which involved the preparation of nanoparticles of palladium by stirring palladium acetate in polyethylene glycol followed by reaction with CoFe_2_O_4_ nanoparticles under ultrasonic irradiation (Scheme 5).

**Scheme 6:**
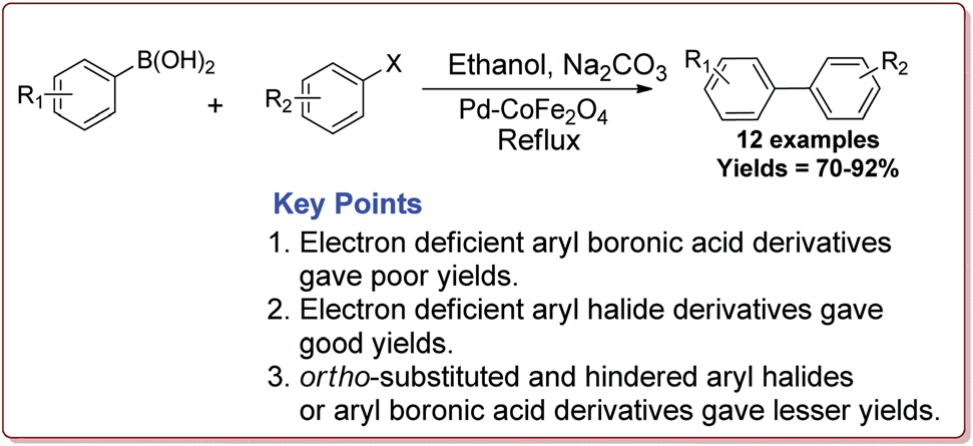
Suzuki coupling reactions catalyzed by Pd–CoFe_2_O_4_ nanocatalyst.

Only 1.6 mol% of the catalyst was required to catalyse the Suzuki coupling reaction between aryl boronic acid and aryl halide derivatives. It was observed that electron deficient aryl boronic acid derivatives gave poor yields whereas the electron deficient aryl halide derivatives gave good yields. Further, *ortho*-substituted and hindered aryl halides or aryl boronic acid derivatives gave lesser yields (Scheme 6). The catalyst was recovered by the external magnet after the completion of the reaction and was used multiple times without affecting its catalytic activity.^122^

**Scheme 7:**
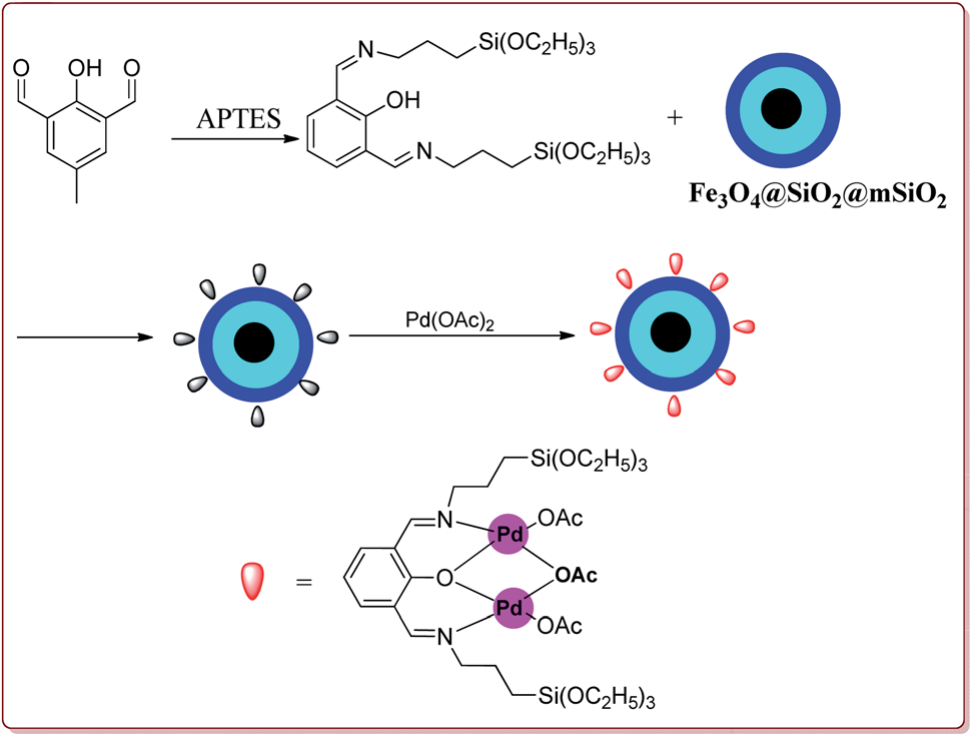
Synthesis of core–shell Fe_3_O_4_@SiO_2_@mSiO_2_-Pd(ii) nanocatalyst.

**Scheme 8:**
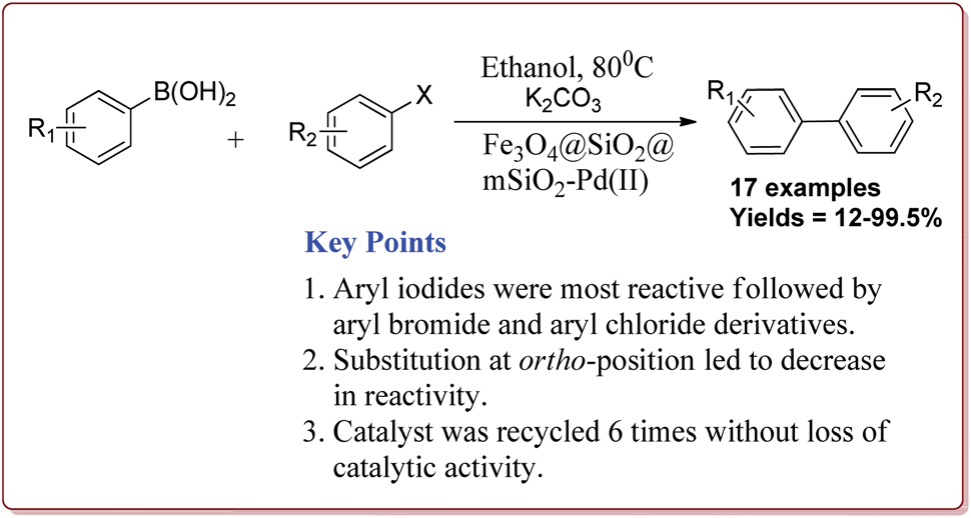
Suzuki coupling reactions catalyzed by core–shell Fe_3_O_4_@SiO_2_@mSiO_2_-Pd(ii) nanocatalyst.

Le *et al.* have reported the cost effective synthesis of Pd(ii) complex functionalized core–shell magnetic mesoporous catalyst Fe_3_O_4_@SiO_2_@mSiO_2_–Pd(ii) having high surface area which exhibited excellent activity against Suzuki–Miyaura coupling reactions (Scheme 7). The catalyst was prepared by the reaction of 3-aminopropyltriethoxysilane (APTES) with mesoporous Fe_3_O_4_@SiO_2_@mSiO_2_ followed by reaction with Pd(OAc)_2_. The best results were obtained in ethanol and K_2_CO_3_ at 80 °C by using 0.5 mol% of the catalyst. In general, aryl iodides were found more reactive than the aryl bromide derivatives and required lesser reaction time. Aryl chloride derivatives gave poor yields with larger reaction time. Further, the presence of electron withdrawing group at *ortho*-position led to decrease in the yield (Scheme 8). The catalyst was recycled for 6 times while retaining the yield of 91%.^123^

**Scheme 9:**
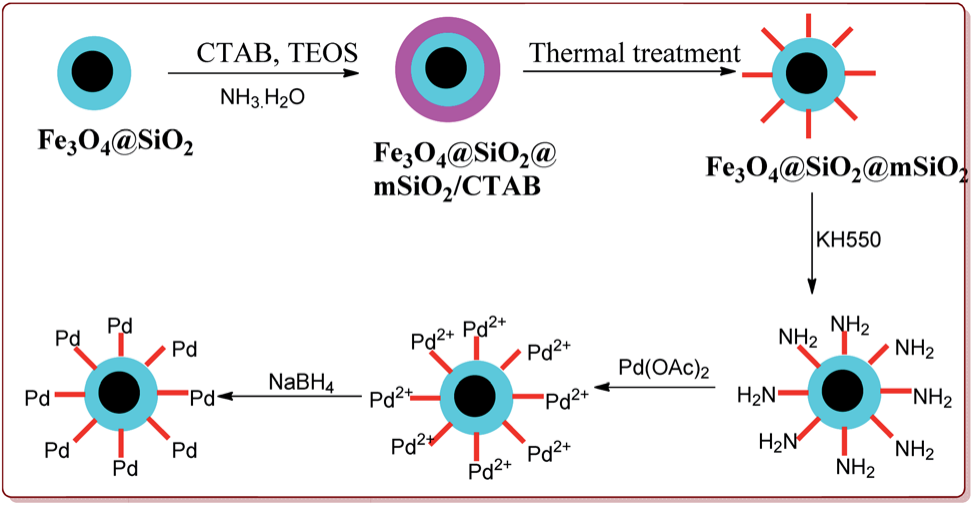
Preparation of immobilized Fe_3_O_4_@SiO_2_@mSiO_2_-Pd(0) nanocatalyst.

Li *et al.* have reported the synthesis of a core–shell magnetic mesoporous Fe_3_O_4_@SiO_2_@mSiO_2_-Pd(0) microspheres having large pore-size mesoporous for catalysing Suzuki coupling reactions. Fe_3_O_4_ nanoparticles were coated on silica by using Stober method followed by reaction with hexadecyltrimethylammonium bromide (CTAB) and TEOS to give Fe_3_O_4_@SiO_2_@mSiO_2_. Further reaction steps involved the reaction with Pd(OAC)_2_ and reduction with NaBH_4_ to give the target catalyst (Scheme 9).

**Scheme 10:**
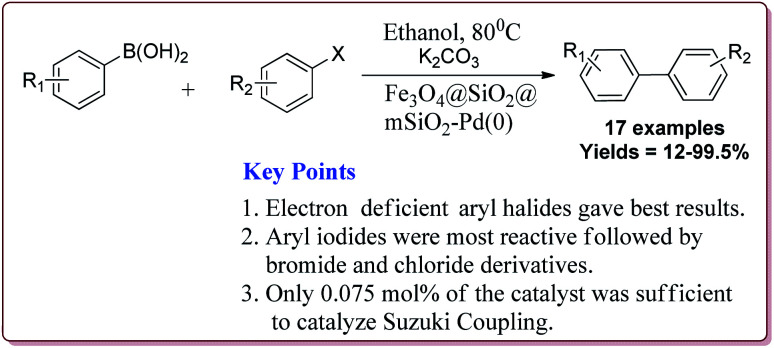
Suzuki reactions catalyzed by Fe_3_O_4_@SiO_2_@mSiO_2_-Pd(0) nanocatalyst.

The catalytic activity of the catalyst was attributed to the dense inner shell which protected the magnetic core and the porous outer shell which provide large surface area for Pd loading which coordinated with the amine-modified core–shell magnetic mesoporous Fe_3_O_4_@SiO_2_@mSiO_2_ microspheres. Iodobenzene was found to give best results followed by bromo and chlorobenzene. Further, electron deficient aryl halide derivatives facilitated the Suzuki coupling (Scheme 10). Interestingly, only 0.075 mol% of the catalyst was sufficient to catalyse the reaction and it could be used six times without losing its catalytic activity.^124^

**Scheme 11:**
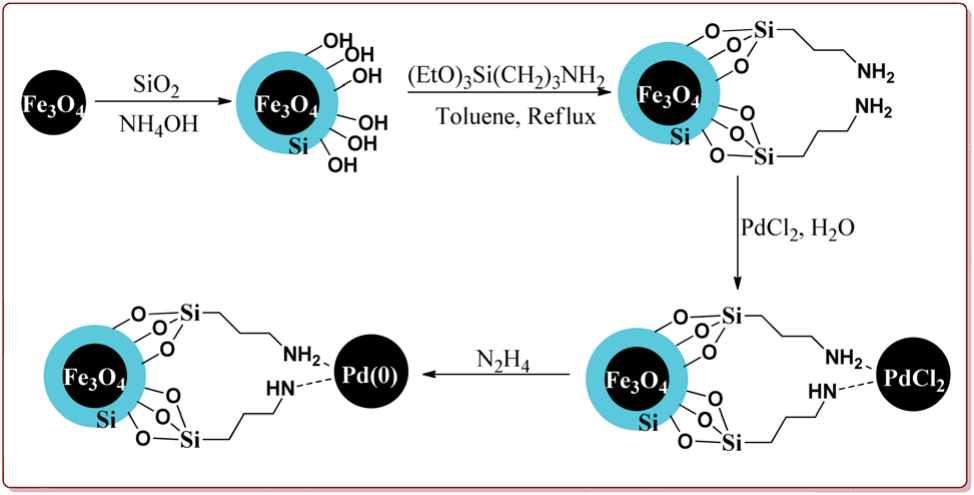
Synthesis of Fe_3_O_4_@SiO_2_–Pd(0) nanocatalyst.

Khazaei *et al.* have reported the synthesis of Fe_3_O_4_@SiO_2_ nanoparticles supported on Pd(O) for catalysing Suzuki coupling reactions. The catalyst was synthesized by using rice husk biomass as a source of biosilica. The synthesis of the desired catalyst involved the preparation of the Fe_3_O_4_ nanoparticles by co-precipitation method followed by reaction with silica in the presence of NH_4_OH. Next steps involved the reaction with 3-(triethoxysilyl)propylamine followed by reaction with PdCl_2_. Last step involved the reaction with N_2_H_4_ to get the desired functionalized Fe_3_O_4_@SiO_2_–Pd(0) nanocatalyst (Scheme 11).

**Scheme 12:**
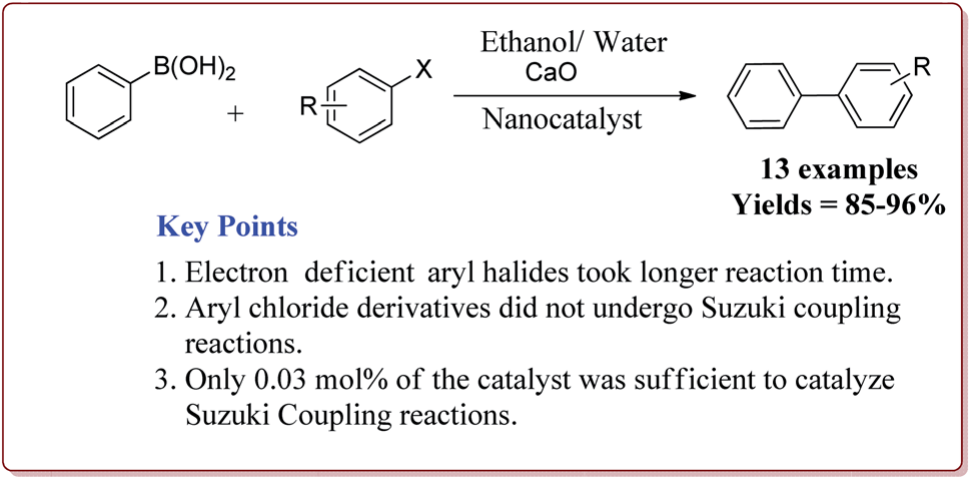
Suzuki coupling reactions of phenyl boronic acid with aryl halides catalyzed by functionalized Fe_3_O_4_@SiO_2_–Pd(0) nanocatalyst.

Only 0.03 mol% of the catalyst was sufficient of carry out Suzuki coupling reaction by using CaO as base in H_2_O/ethanol mixture at high temperature. In general, excellent yields were observed in all the cases. However, electron deficient aryl halides took longer time for reaction completion. Interestingly, aryl chloride did not react under these conditions (Scheme 12). The catalyst was recovered and was reused for 5 times without any loss in its activity.^125^

#### 4.1.2 Heck coupling reaction

**Fig. 6:**
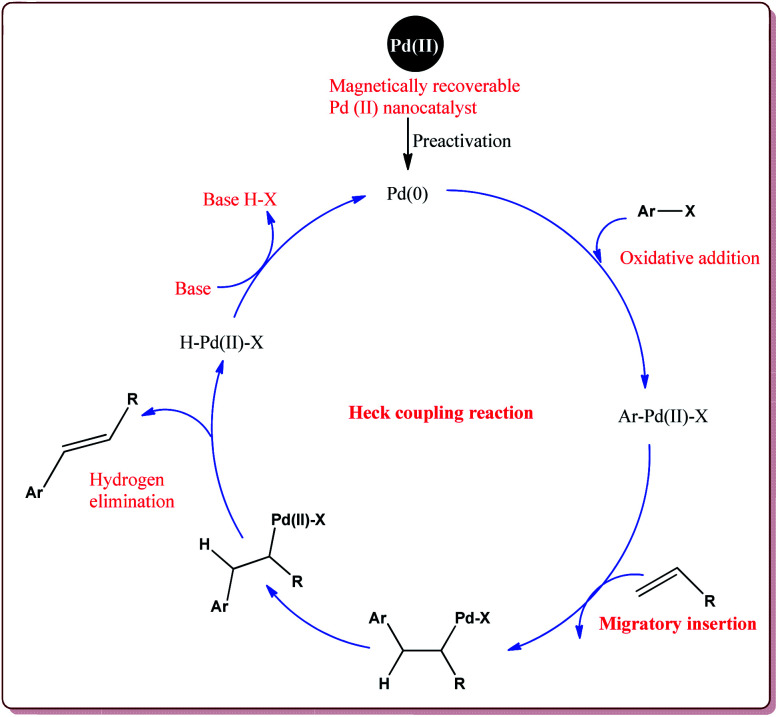
Mechanistic pathway of Heck coupling reaction.

The arylation or vinylation of alkene derivatives was discovered by Mizoroki and Heck in 1970.^126,127^ Heck reaction involving the palladium catalyzed reaction between alkenyl or aryl halide is an another important class of reactions for carbon–carbon bond formation.^128^ This approach has been widely used for the preparation of the biologically active compounds and natural products at small as well as at industrial scale.^129^ Many studies have been reported in literature for the significance and practical importance of this method.^130,131^ The typical catalytic cycle of the Heck coupling reaction is depicted in Fig. 6.

**Scheme 13:**
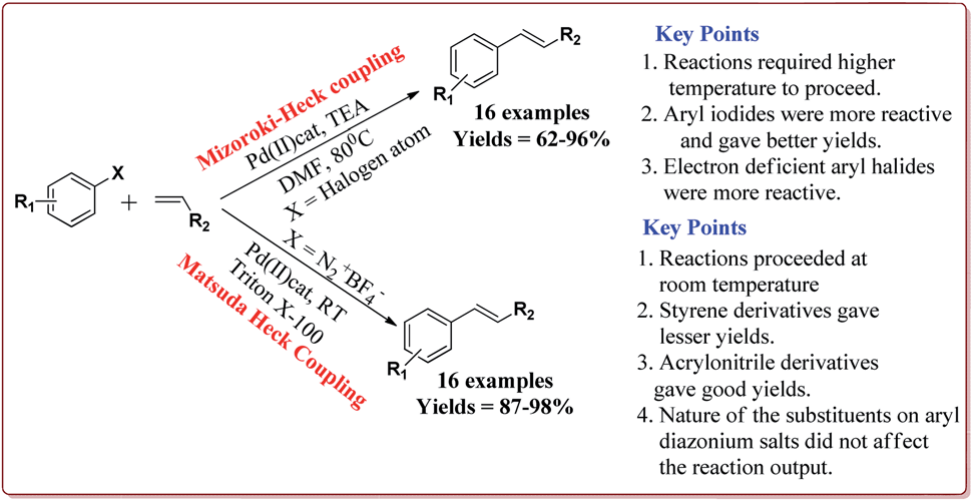
Pd-AcAc-Am-Fe_3_O_4_@SiO_2_ nanocatalyst catalyzed Mizoroki and Matsuda Heck coupling reactions.

Vibhute *et al.* have reported the synthesis of palladium supporting Schiff-base complex immobilized on magnetic nanoparticles, Pd-AcAc-Am-Fe_3_O_4_@SiO_2_, to carry out Mizoroki and Matsuda Heck coupling reactions. The synthesis of Pd-AcAc-Am-Fe_3_O_4_@SiO_2_ nanocatalyst was achieved as shown in Scheme 1. The results suggested that the catalyst tolerate number of functional groups and *trans* products were formed in all the cases in Mizoroki–Heck coupling reactions of aryl halides with terminal olefins at higher temperature. Further, aryl iodides were found to be more active followed by aryl bromides and aryl chlorides. In addition, electron deficient aryl halides were more reactive than the electron rich aryl halides and gave better yields. On the other hand, Matsuda Heck coupling reactions between aryldiazonium slats and terminal alkenes were achieved at room temperature in aqueous medium. Moderate yields were obtained in case of styrene derivatives because of their low reactivity. However, excellent yields were obtained for the reactions of arenediazonium slats and acrylonitrile derivatives. Further, the nature of the group on the aryl diazonium slats did not affect the reaction output (Scheme 13). Only 0.3 mol% of the catalyst was required to complete the reaction and was reused for 6 cycles without loss of its activity.^132^

**Scheme 14:**
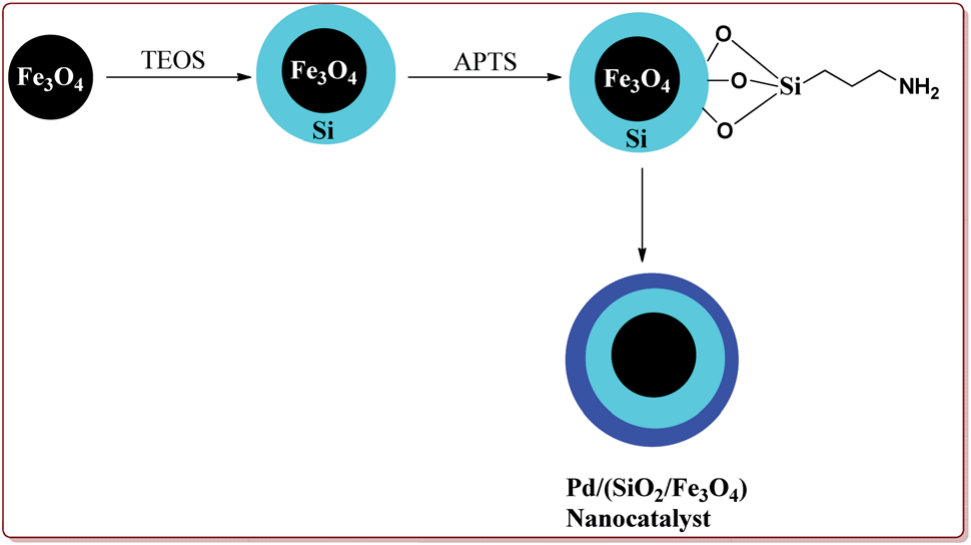
Synthesis of Pd/(SiO_2_/Fe_3_O_4_) nanocatalyst.

**Scheme 15:**
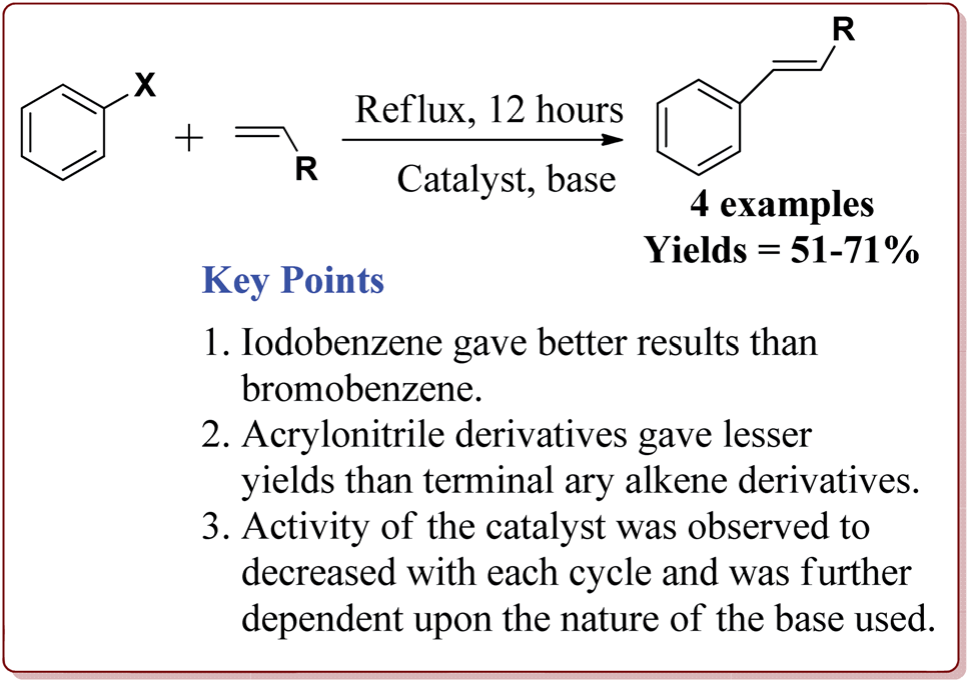
Pd/(SiO_2_/Fe_3_O_4_) catalyzed Heck reaction.

Wang *et al.* have reported the synthesis of palladium catalyst based upon magnetic nanoparticles by using bottom up approach to explore its applicability in Heck coupling of acrylic acid derivatives with aryl halides. The Fe_3_O_4_ nanoparticles were prepared by chemical co-precipitation method which were further reacted with sodium silicate and TEOS to give Fe_3_O_4_@SiO_2_. Further reaction with 3-aminopropyl triethoxysilane (APTS) under vigorous stirring followed by reaction with H_2_PdCl_4_ gave the targeted Pd/(SiO_2_/Fe_3_O_4_) nanocatalyst (Scheme 14). The studies suggested that the aryl iodides gave better yields than the aryl bromides. On the other hand, acrylic acid derivatives gave lesser yield than the terminal aryl alkenes (Scheme 15). Only 30 mg of the catalyst was required for the reaction completion. However, the catalytic activity of the catalyst was found to decreased with each cycle and was further dependent upon the type of the base used.^133^

**Scheme 16:**
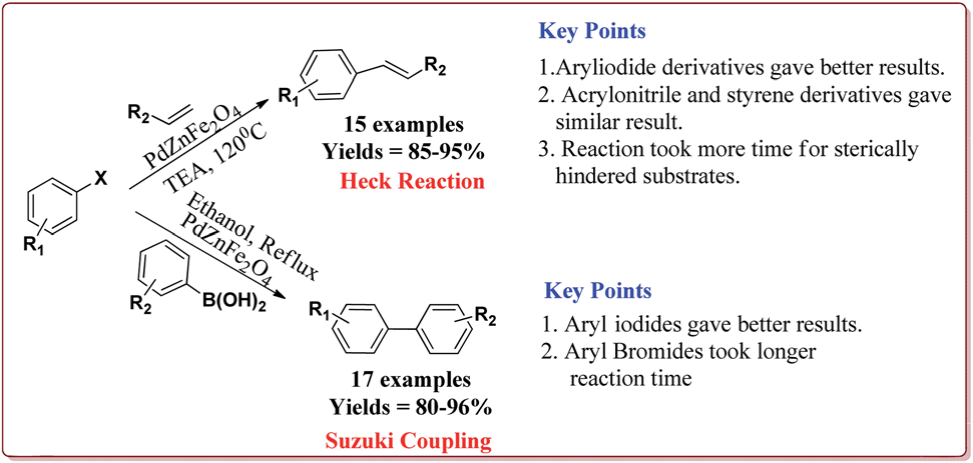
Heck coupling reactions catalyzed by Pd–ZnFe_2_O_4_ nanocatalyst.

Singh *et al.* have reported the synthesis of a super-paramagnetic palladium supporting zinc ferrite nanoparticles by ultrasound assisted co-precipitation method. The scope of the prepared catalyst was studied for Heck and Suzuki reactions of variety of aryl halides and alkene derivatives. In general, aryl iodides were found to be more reactive than bromo derivatives in both the reactions. Both, electron deficient as well as electron rich aryl halides gave similar results. For Heck reactions, acrylic and styrene derivatives gave similar results. However, hindered alkenes took more time for completion of the reactions. Only 4.2 mol% of the catalyst was found sufficient for the completion of the reactions and could be used for 5 times without any loss in its catalytic activity (Scheme 16).^134^

**Scheme 17:**
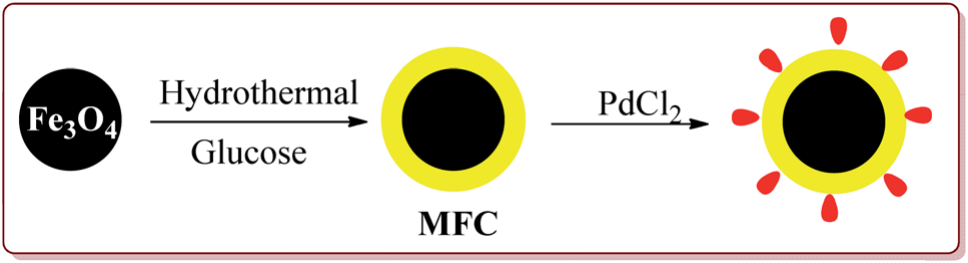
Preparation of Pd/Fe_3_O_4_@C nanocatalyst.

Zhu *et al.* have reported the synthesis of carbon nanocomposite supported Pd nanoparticles (Pd/Fe_3_O_4_@C) in three steps the first step of which involved the preparation of Fe_3_O_4_ nanoparticles by hydrothermal method. Second step involved the reaction of Fe_3_O_4_ nanoparticles with glucose under ultrasonic irradiation in autoclave to get MFC. In the last stage, Pd/Fe_3_O_4_@C catalyst was obtained by deposition–precipitation method under ultrasonication by reaction with PdCl_2_ (Scheme 17).

**Scheme 18:**
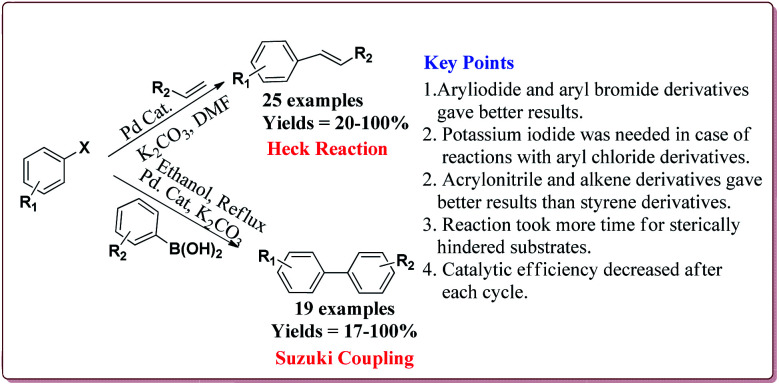
Pd/Fe_3_O_4_@C catalyzed Suzuki and Heck coupling reactions.

The prepared catalyst was used to perform Suzuki and Heck coupling reactions. The studies suggested similar observations for both type of reactions. Aryl iodide and aryl bromide derivatives gave good yields whereas the aryl chloride derivatives gave poor yields. Also, less hindered substrate gave better yields. In case of Heck reaction, acrylates and alkene derivatives gave better results as compared to styrene derivatives. Further, only 30 mg of the catalyst was sufficient to carry out the reactions. However, the yields of the reactions were found to decrease on reusing the catalyst (Scheme 18).^135^

#### 4.1.3 Sonogashira coupling reaction

**Fig. 7:**
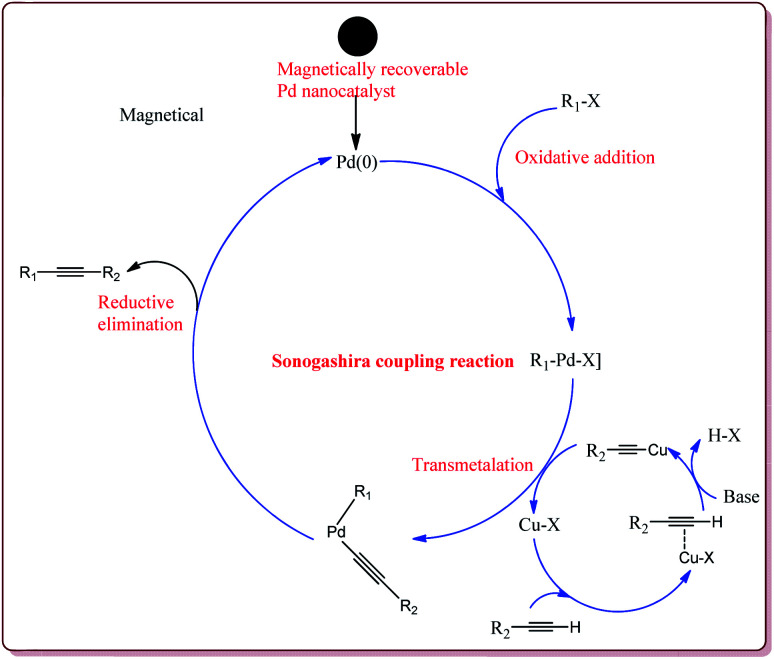
Mechanistic pathway of Sonogashira coupling reaction.

The coupling between aryl, vinyl or pseudo halide derivatives with terminal acetylene derivatives by using Pd(0) catalyst in the presence of amine is called Sonogashira cross-coupling reaction.^136^ It is one of the most important reactions for C(sp^2^)–C(sp) bond formation^137^ and is widely used for its applications in the synthesis of natural products,^138^ agrochemicals and pharmaceuticals,^139^ polymers and nanostructures.^140–143^ Typical catalytic cycle of the Heck coupling reaction is depicted in Fig. 7.

**Scheme 19:**
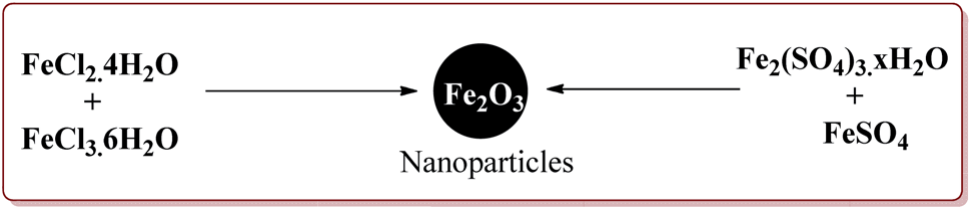
Synthesis of Fe_3_O_4_ nanoparticles.

Firouzabadi *et al.* have reported the ligand free Sonogashira–Hagihara reactions of aryl iodides with terminal alkyne derivatives in ethylene glycol in the presence of Fe_3_O_4_ nanoparticles to give corresponding arylalkyne derivatives. The nanoparticles of Fe_2_O_3_ were prepared either from the reaction of FeCl_2_·4H_2_O and FeCl_3_·6H_2_O or from the reaction of Fe(SO_4_)_3_·*x*H_2_O and FeSO_4_ by literature reported methods (Scheme 19).^144,145^

**Scheme 20:**
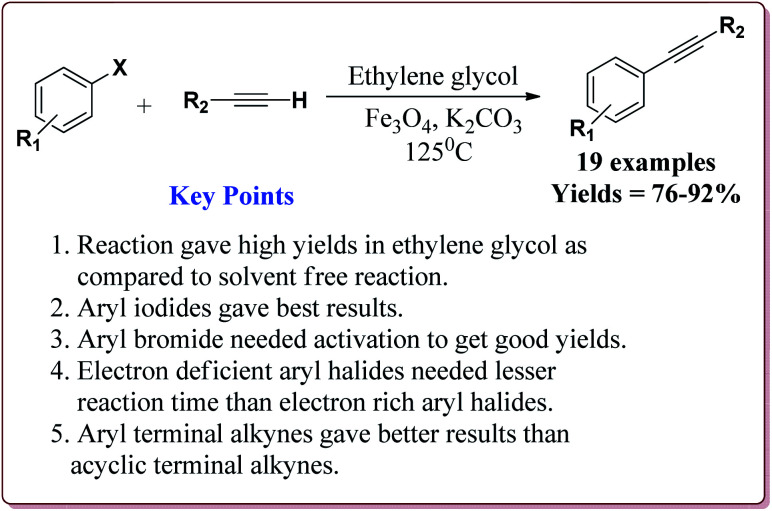
Fe_3_O_4_ nanoparticles catalyzed Sonogashira–Hagihara reactions.

The structure activity relationship studies (SAR) showed that the electron rich aryl halides took longer reaction time as compared to electron deficient aryl halides. Further, aryl iodides were found to be reactive whereas aryl bromide needed activation to get the desired products. Also, the aryl terminal alkene gave better results than the acyclic terminal alkene derivatives (Scheme 20). Only 5 mol% of the catalyst was needed to carry out the reaction and was recycled for 5 cycles without any loss in its catalytic activity.^146^

**Scheme 21:**
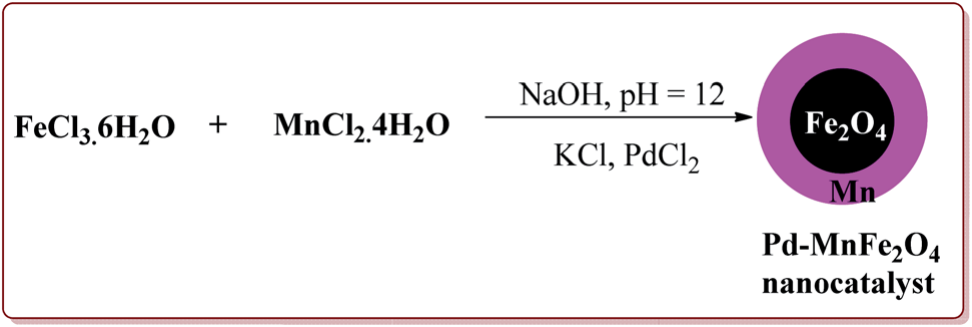
Preparation of Pd–MnFe_2_O_4_ nanocatalyst by one pot sonochemical co-precipitation method.

Jadhav *et al.* have prepared Pd–MnFe_2_O_4_ nanocatalyst by one pot sonochemical co-precipitation method for its application to carry out Sonogashira reactions for the synthesis of symmetric as well as asymmetric alkynes in good to excellent yields *via* decarboxylative coupling of terminal alkynes with arenediazonium salts (Scheme 21).

**Scheme 22:**
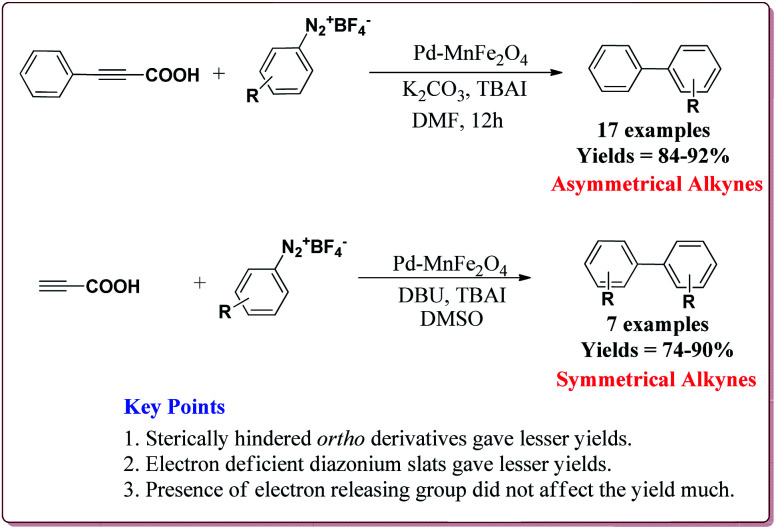
Pd–MnFe_2_O_4_ nanocatalyst catalyzed Sonogashira reactions.

The studies suggested that the sterically hindered *ortho*-substituted diazonium slats gave lesser yields. Also, the electron deficient diazonium slats were found to be less reactive whereas the presence of electron releasing group did not affect the yield much. Only 1 mol% of the catalyst was sufficient to carry out the reaction and was reused for 5 cycles without any appreciable loss in its catalytic activity (Scheme 22).^147^

**Scheme 23:**
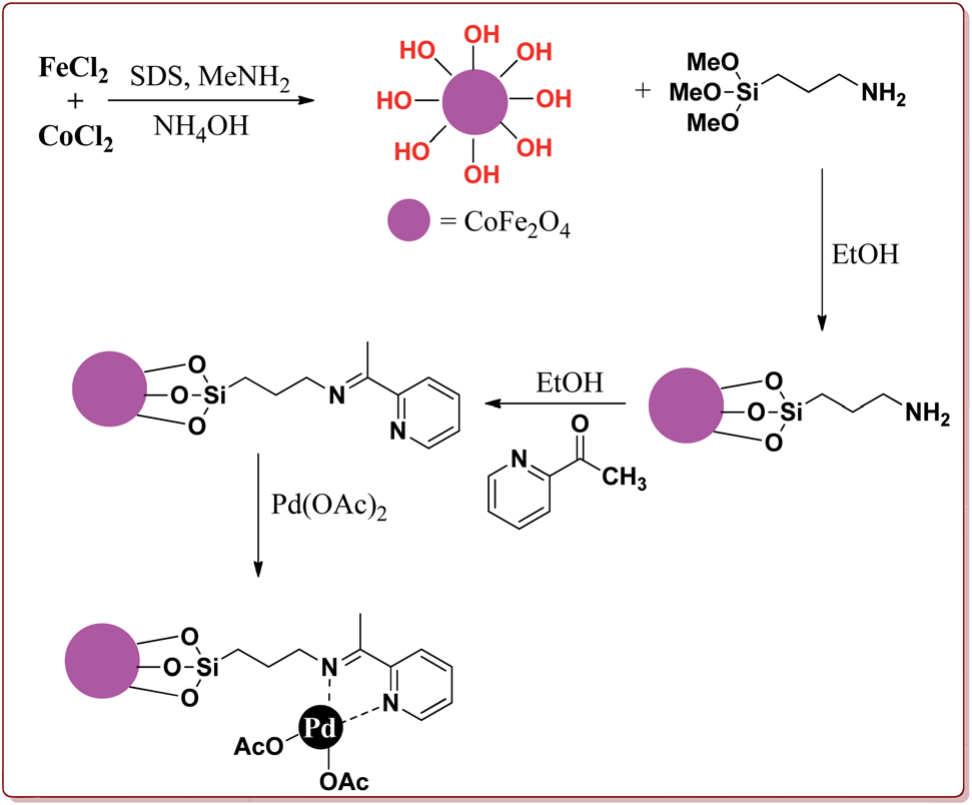
Synthesis of superparamagnetic nanoparticles supported phosphine-free palladium catalyst.

Bui *et al.* have reported the synthesis of superparamagnetic nanoparticles supported phosphine-free palladium catalyst for catalysing the Sonogashira coupling reactions. The target catalyst was prepared by following microemulsion method involving reaction of FeCl_2_ and CoCl_2_ in sodium dodecyl sulphate (SAD) to get superparamagnetic cobalt spinel ferrite nanoparticles which were further reacted with 3-(trimethoxysilyl)propylamine to give amino-functionalized magnetic nanoparticles. These were further reacted with 2-acetyl pyridine followed by reaction with palladium acetate to give the target catalyst (Scheme 23).

**Scheme 24:**
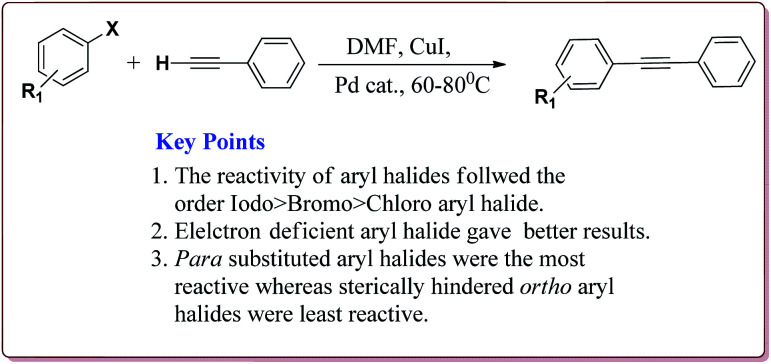
Sonogashira reactions between phenyl acetylene and aryl halides catalyzed by palladium nanocatalyst.

It was observed that the catalyst was able to catalyse the Sonogashira reactions in 0.5 mol% ratio. Also, the aryl iodides were found to be more reactive than arylchlorides which needed the presence of electron withdrawing groups on aryl ring to give the reactions in moderate yields. On the other hand, electron rich aryl halides gave lesser yields. Interestingly the *para* substituted aryl halides gave best yields followed by sterically hindered *meta* and *ortho* isomers. Interestingly, the catalyst could be used for 5 runs without loss of activity if purified in DMF at higher temperature before reuse (Scheme 24).^148^

#### 4.1.4 A_3_ coupling reaction

**Fig. 8:**
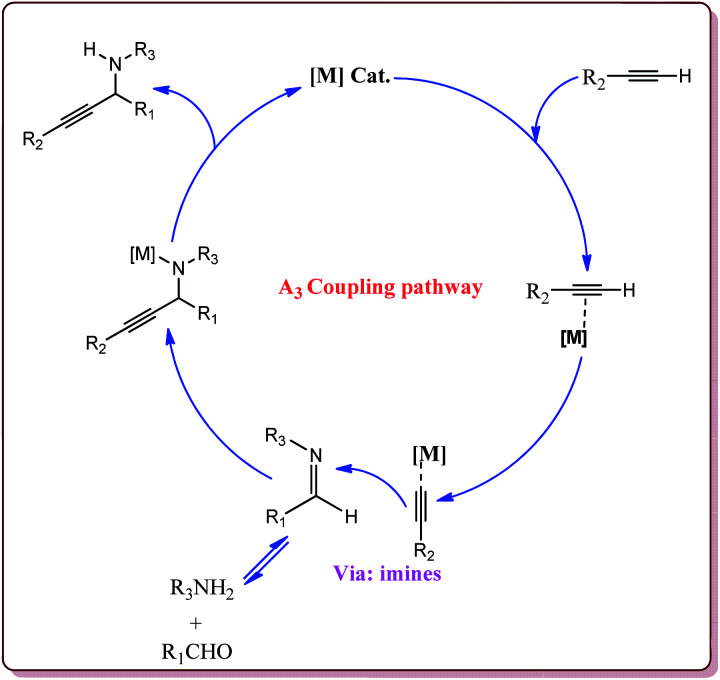
Mechanistic pathway of A_3_ coupling reaction.

A three component reaction between an aldehyde, an amine and an alkyne to give corresponding substituted alkyne derivatives is known as A_3_ coupling. It involves the activation of C–H bond of alkyne by transition metal catalyst for *in situ* generation of metal acetylide which is a common step for Sonogashira and A_3_ coupling reaction. The first example of A_3_ coupling was reported in 1998 by Dyatkin *et al.*^149^ After that, number of synthetic applications of this reaction for the synthesis of heterocyclic compounds, propargylamines, polycyclic pyrroles, 3-benzazepines, imidazole derivatives, lactones, thiazolidines and glyco-conjugates have been reported in literature.^150–153^ Typical mechanism of A_3_ coupling is depicted in Fig. 8.

**Scheme 25:**
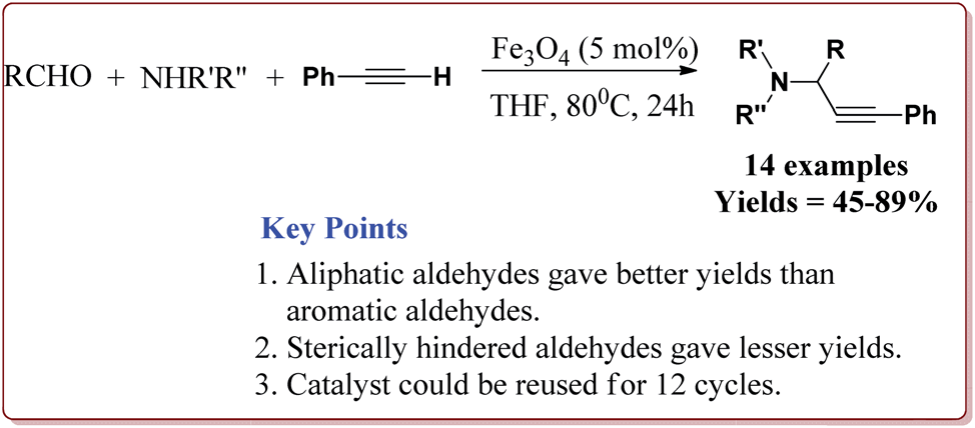
Fe_3_O_4_ nanoparticles catalyzed A_3_ coupling reaction.

Zeng *et al.* have reported the use of Fe_3_O_4_ nanoparticles having size less than 50 nm as a nanocatalyst to carry out the A_3_ coupling reaction between aldehyde, phenylacetylene and amine derivatives. The reaction gave moderate to good yields with various aliphatic and aromatic aldehydes. Aliphatic aldehyde derivatives gave better yields than aromatic aldehydes. Within aliphatic aldehydes, satirically hindered aldehyde derivatives gave lesser yields whereas straight chain aldehydes gave better yields. Also, six membered amine derivatives gave better yields as compared 5 membered amine derivatives. Further, only 5 mol% of the catalyst was necessary to catalyse the reaction and the catalyst could be used for 12 times without any loss in its catalytic activity (Scheme 25).^154^

**Scheme 26:**
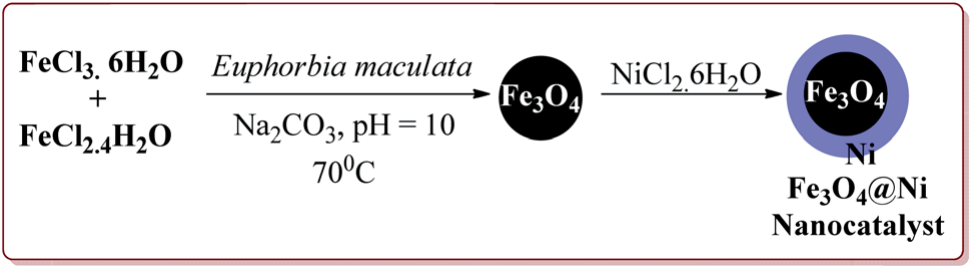
Synthesis of Fe_3_O_4_@Ni nanocatalyst.

Alinezhad *et al.* have reported the biosynthesized Fe_3_O_4_@Ni nanoparticles catalyzed A_3_ coupling and Sonogashira reactions. The catalyst was prepared by a green method the first step of which involved the reaction of FeCl_3_·6H_2_O and FeCl_2_·4H_2_O in *Euphorbia maculata* extracts at 70 °C by maintaining the pH of reaction mass at 10 by Na_2_CO_3_ to give Fe_3_O_4_ nanoparticles followed by with NiCl_2_·6H_2_O in *Euphorbia maculata* extracts at 60 °C to give target nanocatalyst (Scheme 26).

**Scheme 27:**
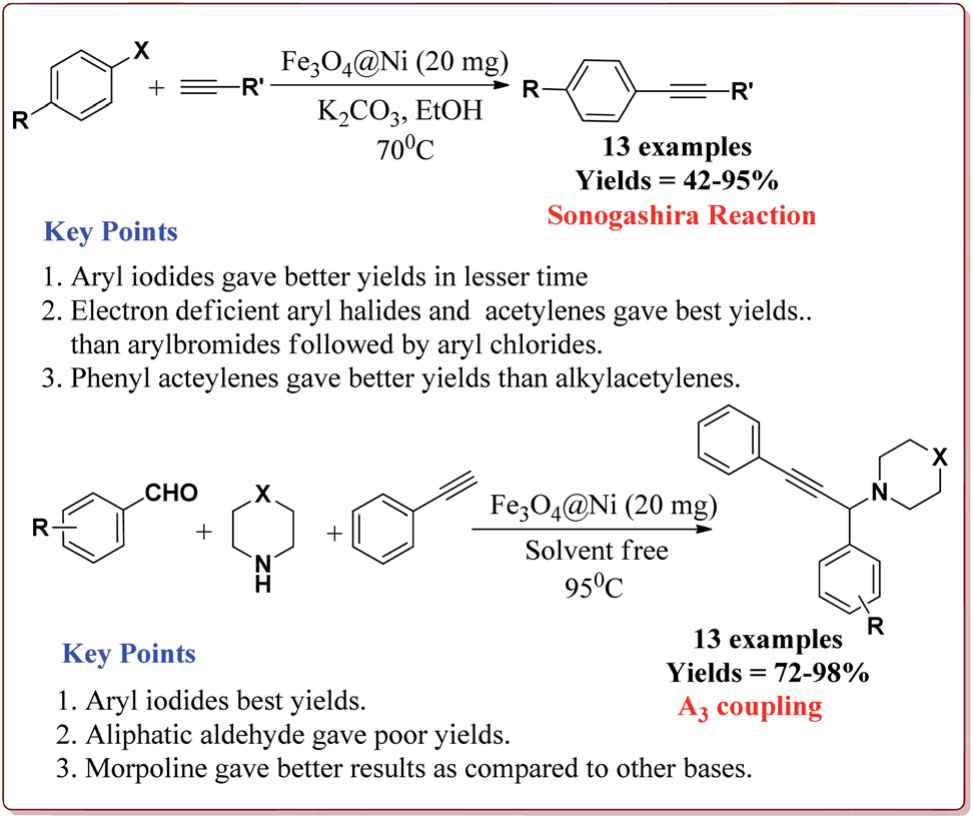
Fe_3_O_4_@Ni catalyzed Sonogashira and A_3_ coupling reactions.

The prepared catalyst gave better yields in case of electron deficient aryl halides and acetylene derivatives for Sonogashira reaction. Further, aryl iodides gave best yields in lesser reaction time on reaction with aryl acetylene derivatives as compared to reaction with alkyl acetylene derivatives. In case of A_3_ coupling, aryl iodides gave better yield in lesser time as compared to aliphatic aldehydes in the presence of morpholine as a base. Interestingly, the catalyst could be used 5 times without loss in its activity after being purified in ethyl acetate (Scheme 27).^155^

### 4.2. Multicomponent reaction

#### 4.2.1 Strecker reaction

**Fig. 9:**
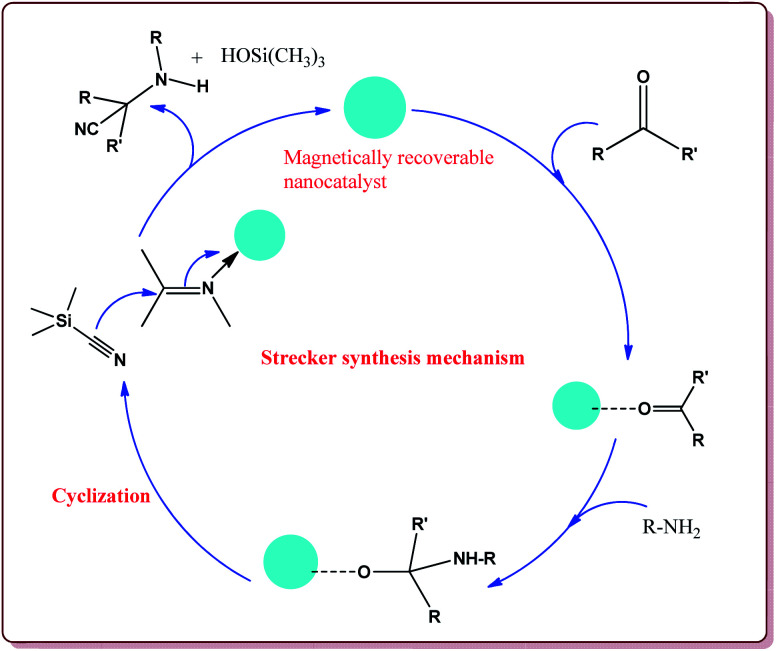
Mechanistic pathway of Strecker synthesis.

The reaction between an aldehyde derivative and ammonium chloride in the presence of potassium cyanide and ammonia to afford α-amino acid derivative is commonly known as Strecker synthesis. It was first reported by Adolf Strecker in 1850.^156^ Since then, significant amount of the work has been reported in literature for the application of this reaction for the preparation of various racemic as well as chiral α-amino acids, α-amino nitriles and heterocyclic derivatives.^157–163^ Typical mechanism of Strecker synthesis is depicted in Fig. 9.

**Scheme 28:**
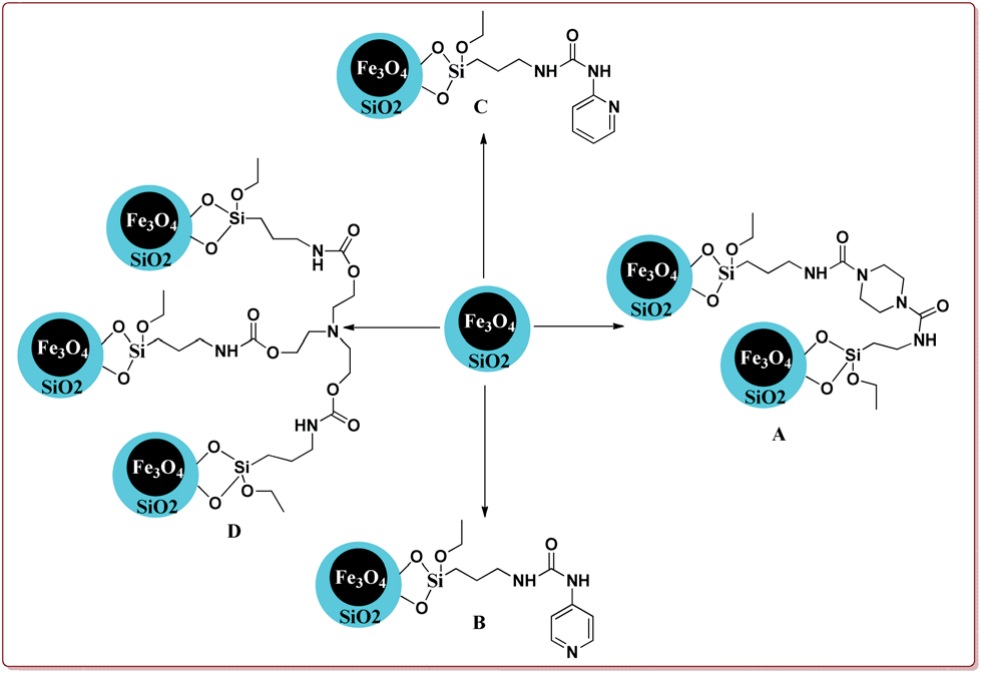
Preparation of novel urea and urethane palladium nanocatalysts.

Baghery *et al.* have reported urea and urethane based four novel magnetic nanocatalysts for the synthesis of α-aminonitriles under solvent free conditions at moderate temperature of 50 °C. The catalysts were prepared by the reaction of Fe_2_O_3_@SiO_2_ nanoparticles with the corresponding urea and urethane derivatives in ethanol under ultrasonic vibrations (Scheme 28).

**Scheme 29:**
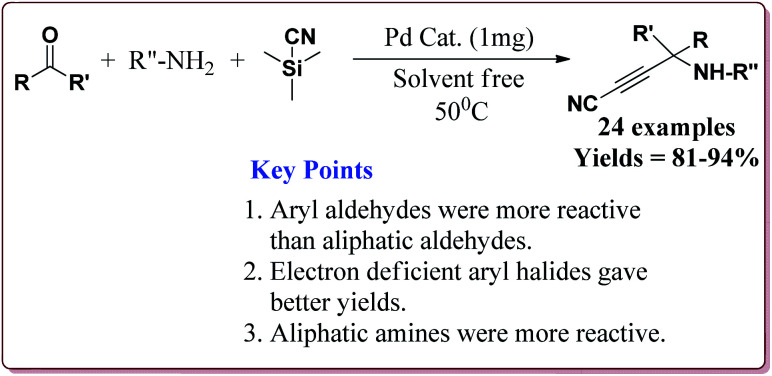
Synthesis of α-aminonitriles derivatives by using urea and urethane based four novel magnetic nanocatalysts.

All the prepared catalysts were efficient in catalysing the Strecker synthesis. Catalyst A was found to be slightly more active followed by **B**, **C** and D. Also, aromatic aldehydes were found to be more reactive than aliphatic aldehydes with electron deficient aryl aldehyde derivatives giving better results than the electron rich aryl aldehydes. Further, aliphatic amines reacted in lesser time under Strecker conditions due to higher nucleophilicity. Only 1 mg of each catalyst was active enough to catalyse the reaction and could be used for 7 times without loss in catalytic activity (Scheme 29).^164^

**Scheme 30:**
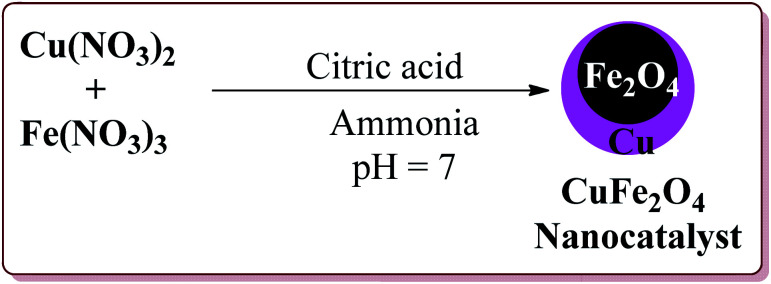
Preparation of CuFe_2_O_4_ nanocatalyst by citrate gel precursor method.

Gharib *et al.* have achieved the synthesis of α-aminonitriles using copper ferrite (CuFe_2_O_4_) nanocatalyst in one pot by the reaction of various aldehyde derivatives, amines and trimethylsilyl cyanide at room temperature by using water as a solvent. The catalyst was synthesized by citrate gel precursor method by reacting copper nitrate and iron nitrate in citric acid solution at pH = 7 maintained by adding ammonia at higher temperature (Scheme 30).^165^

**Scheme 31:**
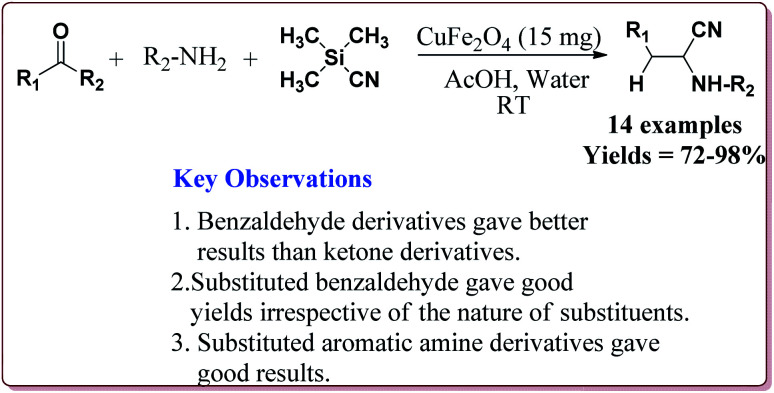
CuFe_2_O_4_ nano catalyst catalyzed preparation of α-amino nitrile derivatives.

It was observed that the reaction of ketone derivatives proceeded with lesser yields as compared to the benzaldehyde derivatives which gave excellent yields irrespective of the type of substitution on the aromatic ring system. Also, the substituted aryl amines gave good results for the formation of the products (Scheme 31).^166^

#### 4.2.2 Biginelli reaction

**Fig. 10:**
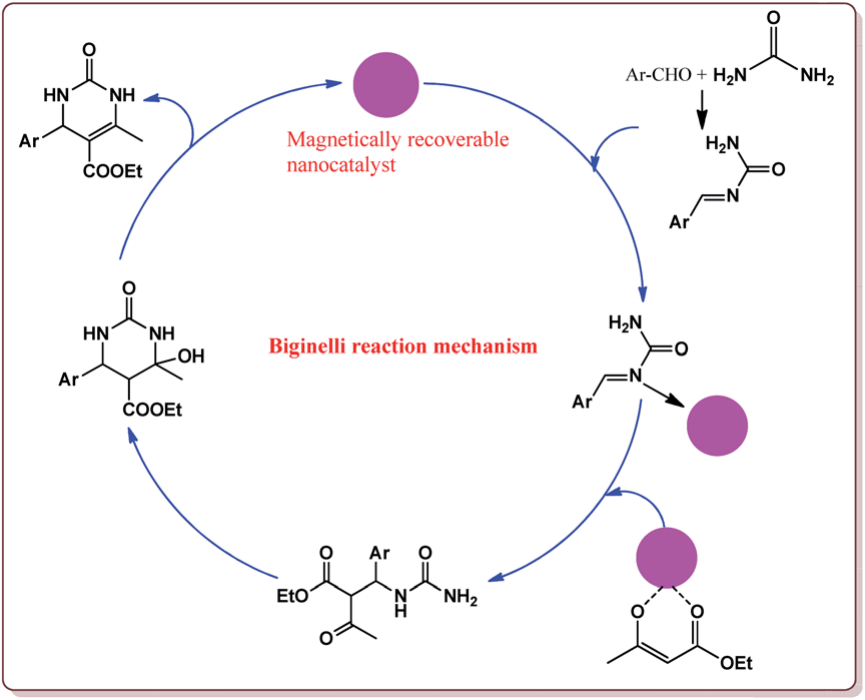
Mechanistic pathway of Biginelli reaction.

Biginelli reaction is one of the classical multicomponent reactions used for the synthesis of heterocyclic derivatives by three component condensation reaction between aldehydes, β-ketoesters and urea or thiourea moiety.^167^ This reaction has found its applications for the synthesis of various biological active compounds^88,168,169^ and many reviews have been reported in literature for its synthetic applications.^170–172^ This reaction has also been used for the asymmetric synthesis of various derivatives of industrial use.^173^ Typical mechanism of Biginelli reaction is depicted in Fig. 10.

**Scheme 32:**
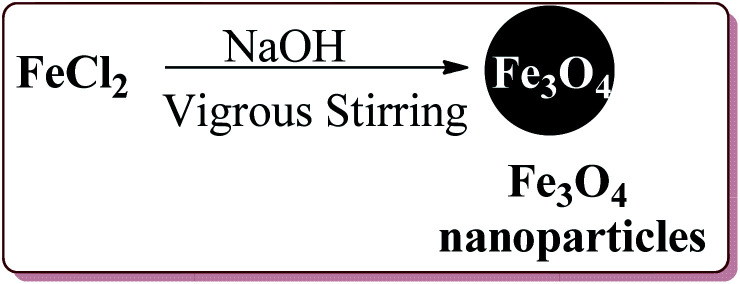
Preparation of Fe_3_O_4_ nanocatalyst.

Nasr-Esfahni *et al.* have reported the use of Fe_3_O_4_ nanoparticles for the synthesis of 3,4-dihydropyrimidin-2(1*H*)-ones by using one pot Biginelli condensation of aromatic aldehydes, urea or thiourea and β-dicarbonyl derivatives under solvent free conditions. The Fe_3_O_4_ nanoparticles were prepared by the reaction of FeCl_2_ with NaOH under vigorous stirring (Scheme 32).

**Scheme 33:**
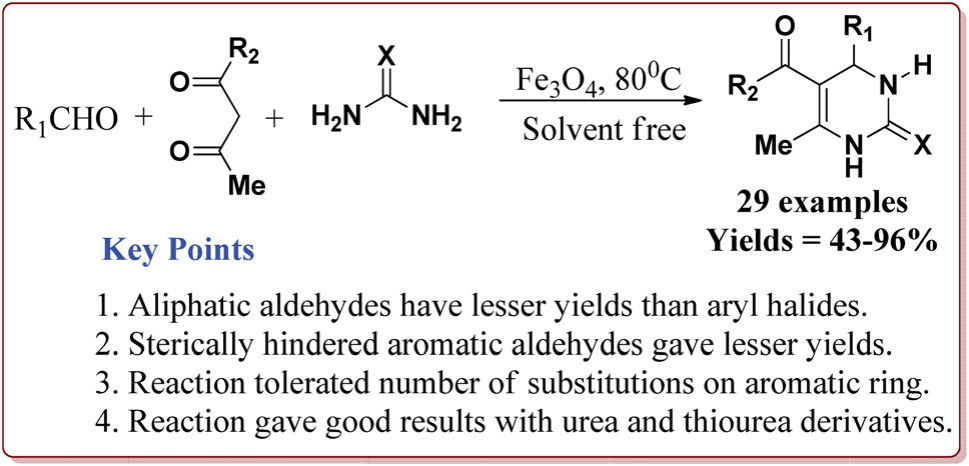
Fe_3_O_4_ nanocatalyst catalyzed Biginelli reactions.

The reaction was well tolerated with various aromatic aldehyde derivatives and gave good yields whereas aliphatic aldehydes gave poor yields. In addition, sterically hindered *ortho* substituted aryl halides gave lesser yields. Also, the reaction gave good results with urea as well as thiourea derivatives and only 20 mol% of the catalyst was sufficient to catalyse the reaction (Scheme 33).^174^

**Scheme 34:**
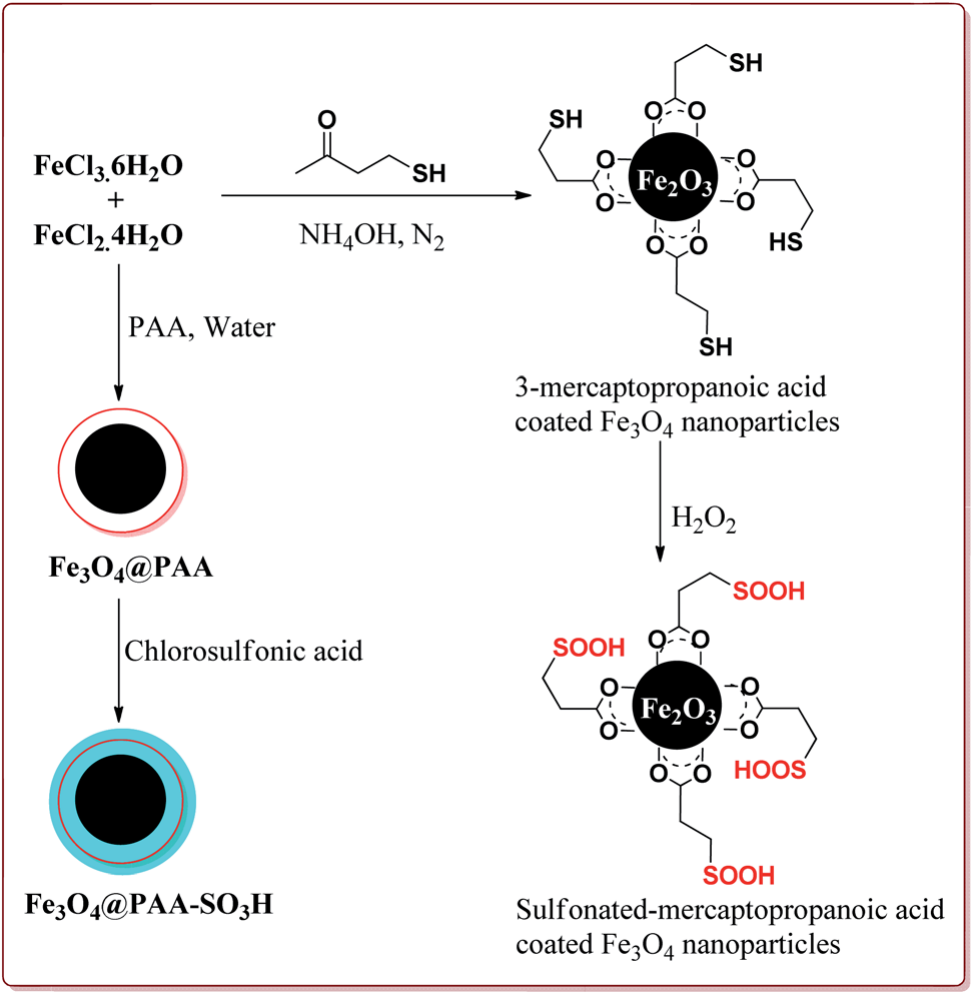
Synthesis of sulfonated-mercaptopropanoic acid coated Fe_3_O_4_ nanoparticles.

**Scheme 35:**
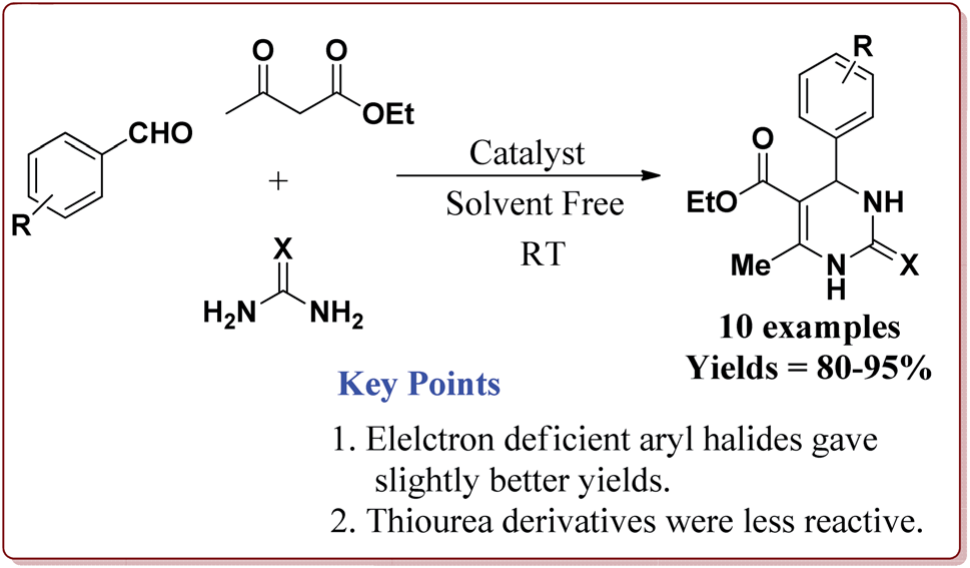
Preparation of 3,4-dihydropyrimidin-2(1*H*)-ones by using sulfonated-mercaptopropanoic acid coated Fe_3_O_4_ nanocatalyst.

Zamani *et al.* have reported the synthesis of sulfonated-mercaptopropanoic acid coated Fe_3_O_4_ nanoparticles to carry out Biginelli reaction for the preparation of 3,4-dihydropyrimidin-2(1*H*)-ones in one pot synthesis. The catalyst was prepared by the reaction of FeCl_3_·6H_2_O and FeCl_2_·4H_2_O followed by the reaction with 3-mercaptopropanoic acid in the first step to give 3-mercaptopropanoic acid coated Fe_3_O_4_ nanoparticles followed by oxidation of terminal thiol groups to sulfonic acid groups by H_2_O_2_ in the second step (Scheme 34). The presence of electron releasing groups on aromatic aldehyde led to slightly decrease in the yield whereas the electron withdrawing groups led to slightly increase in the yields as compared to benzaldehyde. In addition, the thiourea derivatives gave lesser yields as compared to urea derivatives. Further, 60 mg of the catalyst was sufficient to catalyse the reaction and could be used six times effectively (Scheme 35).^175^

In one of the other communications, Zamani *et al.* have reported the synthesis of sulfonated-phenylacetic acid coated Fe_3_O_4_ nanoparticles (Fe_3_O_4_@PAA-SiO_3_H) as a novel nanocatalyst for carrying of Biginelli reaction. The first step of catalyst preparation involved the reaction of FeCl_3_·6H_2_O and FeCl_2_·4H_2_O with phenylacetic acid at pH = 11 followed by sulfonation step by reaction with chlorosulfonic acid to give target catalyst which gave the similar results (Schemes 34 and 35).^176^

**Scheme 36:**
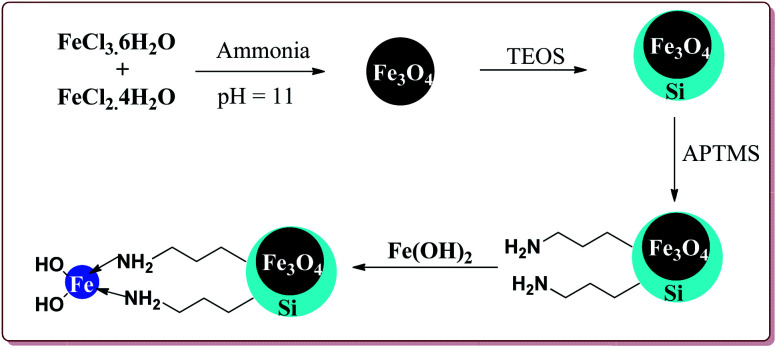
Preparation of Fe_3_O_4_@SiO_2_-APTMS-Fe(OH)_2_ nanocatalyst.

Sheykhan *et al.* have reported the use of Fe_3_O_4_@SiO_2_-APTMS-Fe(OH)_2_ nanoparticles as an efficient catalyst for Biginelli reaction. The catalyst was prepared by reacting FeCl_3_·6H_2_O and FeCl_2_·4H_2_O in ammonia followed by reaction with tetraethyl orthosilicate (TESO) at high temperature to afford Fe_3_O_4_@SiO_2_. Next step involved the reaction with 3-aminopropyltrimethoxysilane (APTMS) and Fe(OH)_2_ to give target catalyst (Scheme 36).

**Scheme 37:**
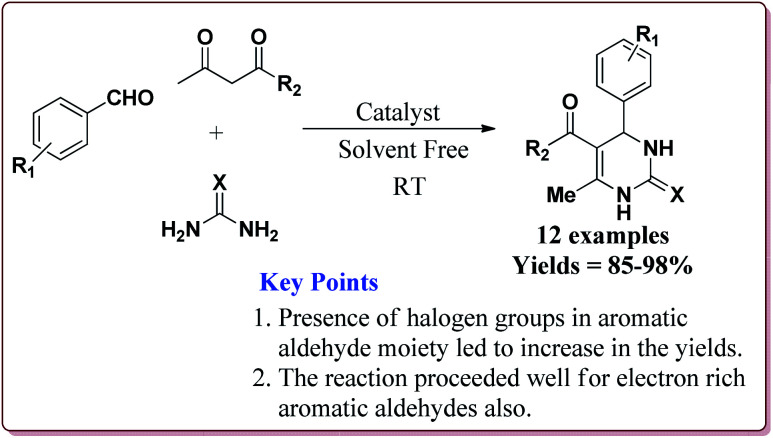
Fe_3_O_4_@SiO_2_-APTMS-Fe(OH)_2_ catalyzed Biginelli reactions.

0.43 mol% of the catalyst could catalyse the Biginelli reaction at high temperature. The presence of halogen group on aromatic aldehyde moiety led to increase in the yields. Interestingly, electron rich aromatic aldehydes also gave good results under these conditions (Scheme 37).^177^

#### 4.2.3 Hantzsch synthesis

**Fig. 11:**
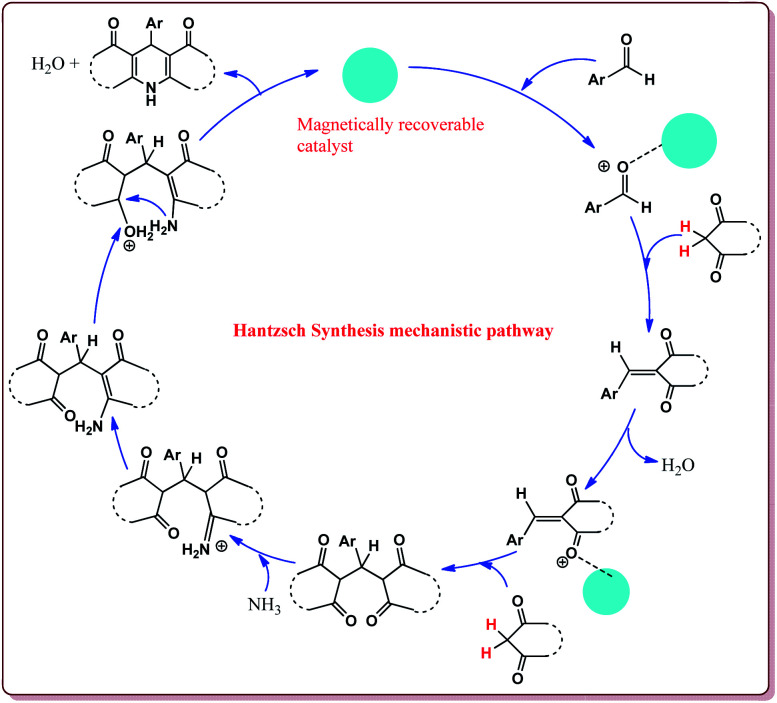
Mechanistic pathway of Hantzsch reaction.

Hantzsch synthesis is one of the most important reaction for the synthesis of 1,4-dihydropyridines (1,4-DHPs) by the reaction of an aldehyde derivative with two equivalents of β-keto ester in the presence ammonia source like ammonium acetate. 1,4-Dihydropyridines (1,4-DHPs) exhibit significant biological as well as medicinal activity.^178–181^ This reaction was first reported in 1882 by Arthur Hantzsch.^182^ Various methods are reported in literature for the use of alternate catalysts to improve the Hantzsch synthesis towards greener chemistry.^183–187^ Mechanism of Hantzsch synthesis is depicted in Fig. 11.

**Scheme 38:**
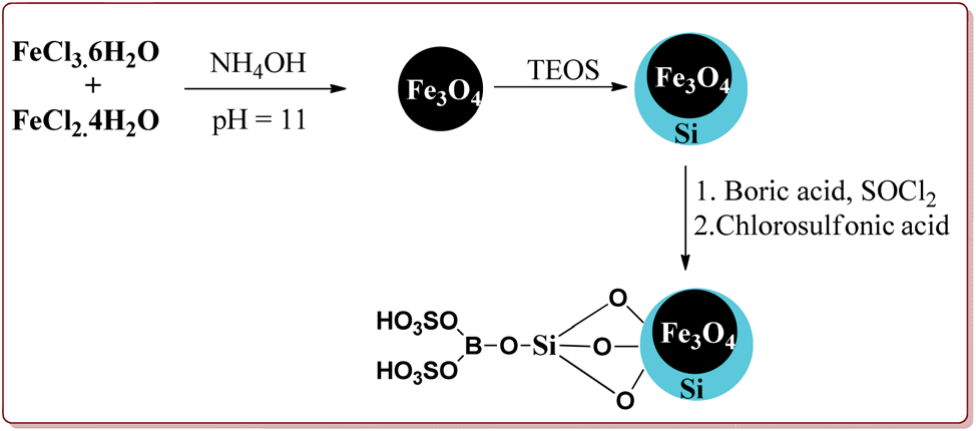
Synthesis of sulfated boric acid–silica-coated magnetite nanoparticles.

Azizi *et al.* have reported magnetically separable sulfated boric acid functionalized nanoparticles as a catalyst to carry out Hantzsch synthesis. Catalyst was prepared by the reaction of Fe_3_O_4_@SiO_2_ with boric acid in the presence of thionyl chloride to get boric acid–silica-coated magnetite nanoparticles which were further reacted with chlorosulfonic acid to afford sulfated boric acid–silica-coated magnetite nanoparticles (Scheme 38).

**Scheme 39:**
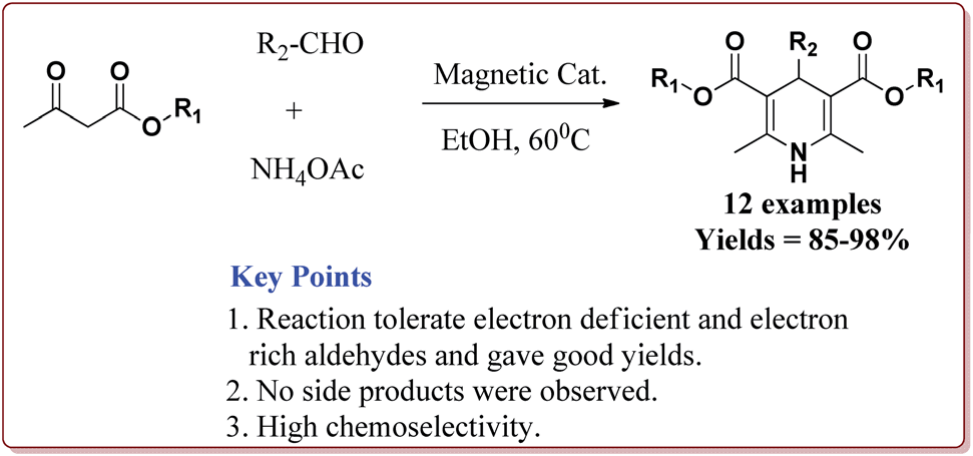
Sulfated boric acid–silica-coated magnetite nanoparticles catalyzed Hantzsch synthesis.

The prepared catalyst was found to very efficient in catalysing the Hantzsch reaction of electron rich as well as electron deficient aldehyde derivatives and gave good yields with high chemo selectivity. No side products were observed during the reactions and only 10 mg of the catalyst was sufficient to catalyse the reaction (Scheme 39).^188^

**Scheme 40:**
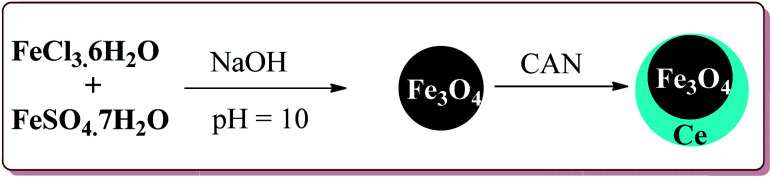
Preparation of magnetite–ceria (Nanocat-Fe–Ce) nanocatalyst.

Gawande *et al.* have reported the applications of magnetite–ceria (Nanocat-Fe–Ce) nanocatalyst for the synthesis of 1,4-dihydropyridines at room temperature by using greener protocol. Fe_3_O_4_ nanoparticles were prepared by already known method and were further reacted with ceric ammonium nitrate (CAN) at pH = 12 to get Nanocat-Fe–Ce catalyst (Scheme 40).

**Scheme 41:**
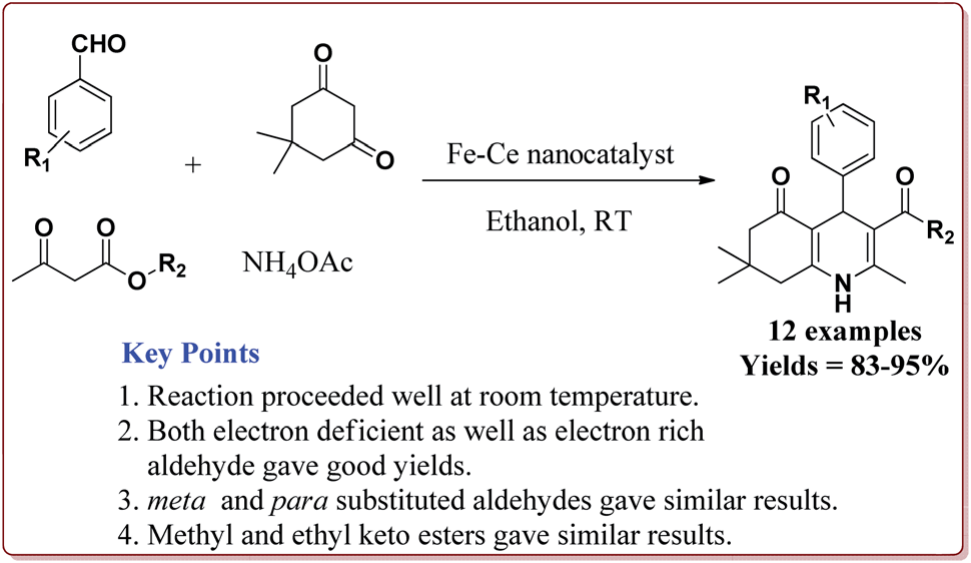
Synthesis of 1,4-dihydropyridines catalyzed by Fe–Ce nanocatalyst.

Only 5.22 mol% of the catalyst was sufficient to catalyze the reaction at room temperature and gave good yields for electron deficient as well as electron rich aldehyde derivatives. Further, both methyl as well as ethyl keto esters afforded good yields. The catalyst could be used six times without loss in its catalytic activity (Scheme 41).^189^

**Scheme 42:**
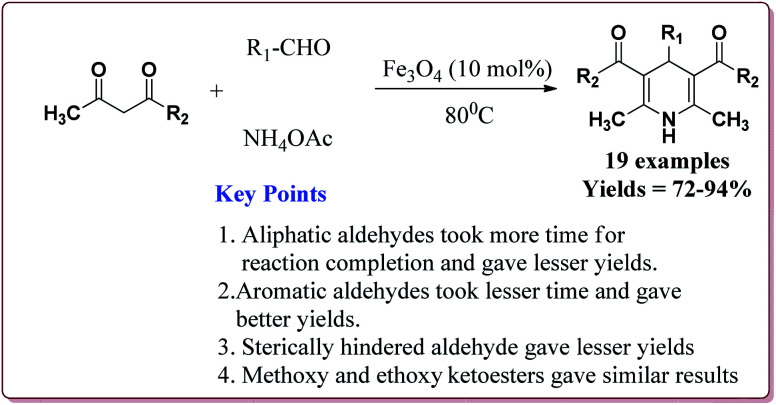
Fe_3_O_4_ nanoparticles catalyzed synthesis of 1,4-dihydropyridine derivatives.

Nasr-Esfahani *et al.* have reported the synthesis of 1,4-dihydropyridines derivatives by using Fe_3_O_4_ nanocatalyst which was prepared by reported method.^190^ Typical procedure involved the reaction of alkyl or aryl aldehyde derivatives, β-dicarbonyl derivatives and ammonium acetate at 80 °C in the presence of 10 mol% of the catalyst. In general, aromatic aldehydes took lesser reaction time than the aliphatic aldehydes and gave better yields. Also, the substitution on the phenyl ring of the aromatic aldehyde led to decrease in the yields. On the other hand, both methyl and ethyl keto ester gave similar results (Scheme 42).^191^

**Scheme 43:**
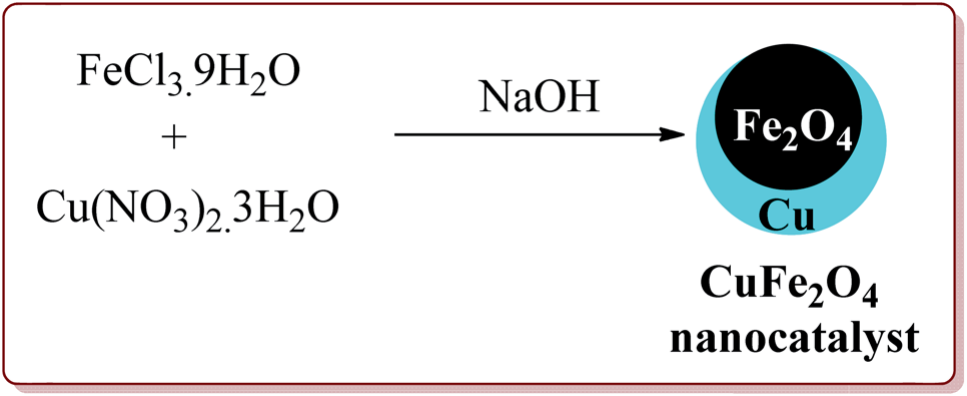
Preparation of CuFe_2_O_4_ nanocatalyst.

Naeimi *et al.* have reported one pot four components synthesis of pyrido[2,3-*d*:6,5-*d*′]dipyrimidines in water by the use of CuFe_2_O_4_ nanoparticles as catalyst which was prepared by co-precipitation method by the reaction of FeCl_3_·9H_2_O and Cu(NO_3_)_2_·3H_2_O in the presence of sodium hydroxide (Scheme 43).

**Scheme 44:**
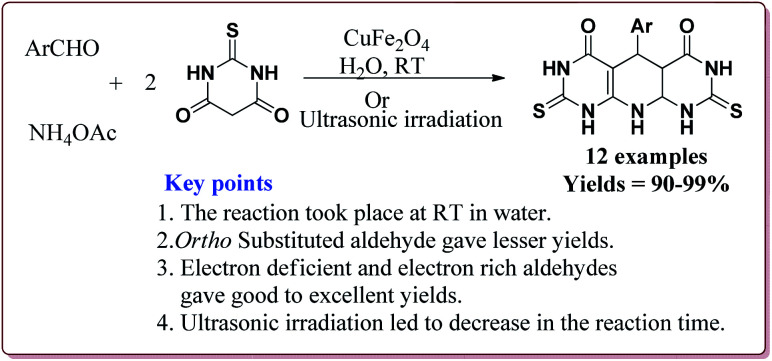
CuFe_2_O_4_ catalyzed synthesis of pyrido[2,3-*d*:6,5-*d*′]dipyrimidines.

The prepared catalyst gave good to excellent yields in case of electron rich and electron deficient aldehyde derivatives. Interestingly, the reaction proceeded well in water at room temperature by using only 10 mol% of the catalyst which could be used for four cycles (Scheme 44).^192^ In an another reported method, Naeimi *et al.* have reported the similar type of reactions by using the same catalyst under ultrasonic irradiation which resulted in decrease in the reaction time for the formation of the products.^193^

#### 4.2.4 Pyrane synthesis

Pyrane or oxine derivatives are monocyclic six-member oxygen-containing heterocyclic, non-aromatic ring compounds. The first reported pyrane derivatives were developed in 1962 by using pyrolysis reactions.^134^ Pyrane derivatives have found their applications as fragrances and flavors,^194^ possess antifungal and antibacterial activity,^195^ anticancer activity^196^ and are thus of industrial as well as medicinal importance.

**Scheme 45:**
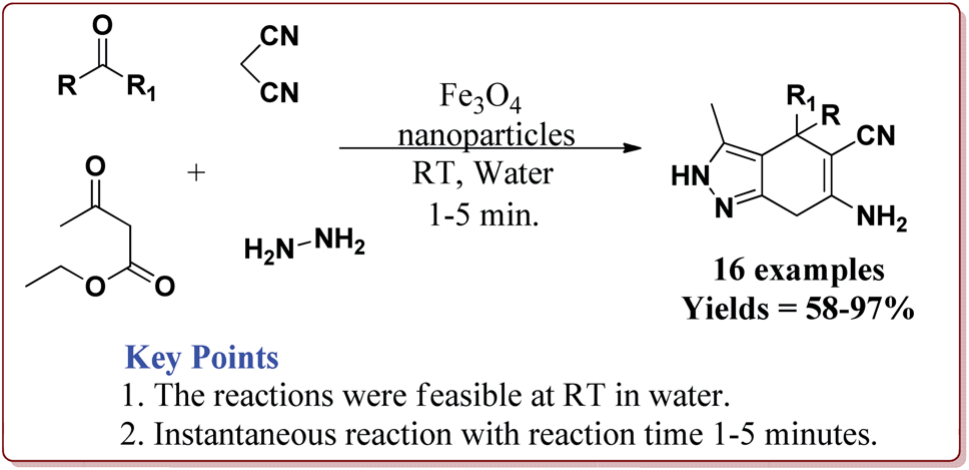
Preparation of pyranopyrazole derivatives by using Fe_3_O_4_ nanoparticles.

Aleem *et al.* have reported the synthesis of pyranopyrazole derivatives in one pot four component reaction of hydrazine hydrate, ethyl acetoacetate, malononitrile and a carbonyl derivative by using Fe_3_O_4_ nanoparticles as catalyst at room temperature by using water as a solvent. Interestingly, only 6 mol% of the catalyst was required for the completion of the reaction in only 1–5 minutes. Further, the catalyst could be used 14 times after purification from ethanol with slight loss of activity after 7^th^ cycle (Scheme 45).^197^

**Scheme 46:**
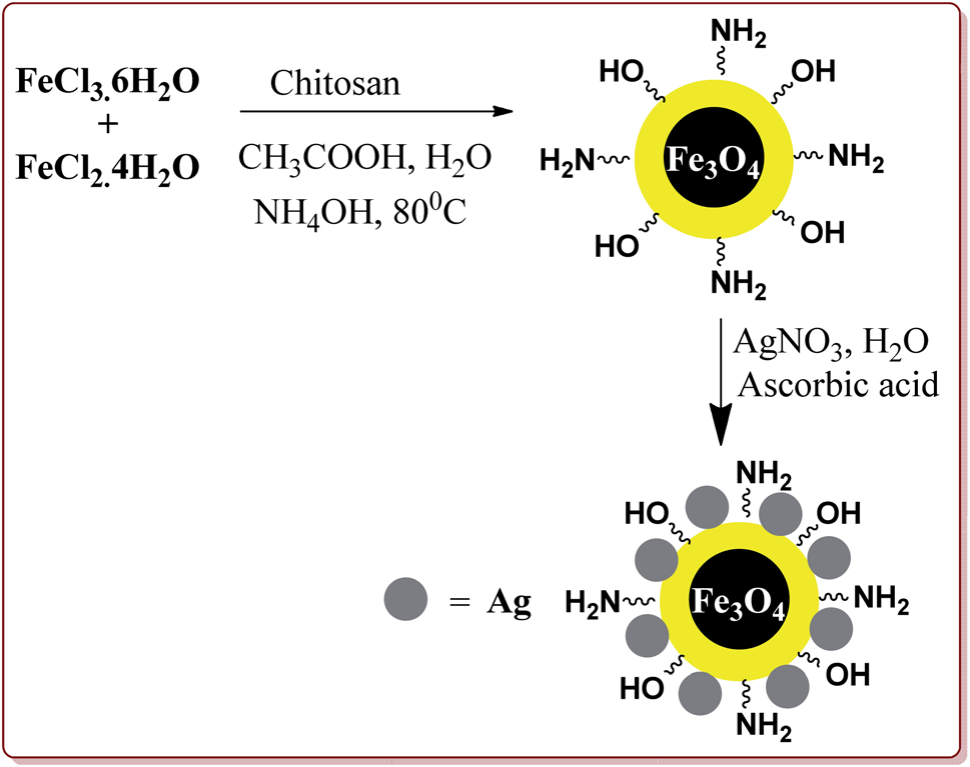
Synthesis of Fe_3_O_4_/CS-Ag nanocatalyst.

Mohammadi *et al.* have reported silver nanoparticles coated magnetic chitosan (Fe_3_O_4_/CS-Ag) catalyzed one pot three component green synthesis of tetrahydrobenzo[α]xanthene-11-ones. The first step of the catalyst preparation involved the reaction of FeCl_3_·6H_2_O and FeCl_2_·4H_2_O in the presence of chitosan to give chitosan-coated magnetic nanoparticles (Fe_3_O_4_/CS) followed by the reaction with silver nitrate in an ultrasonic bath to finally give Fe_3_O_4_/CS-Ag (Scheme 46).

**Scheme 47:**
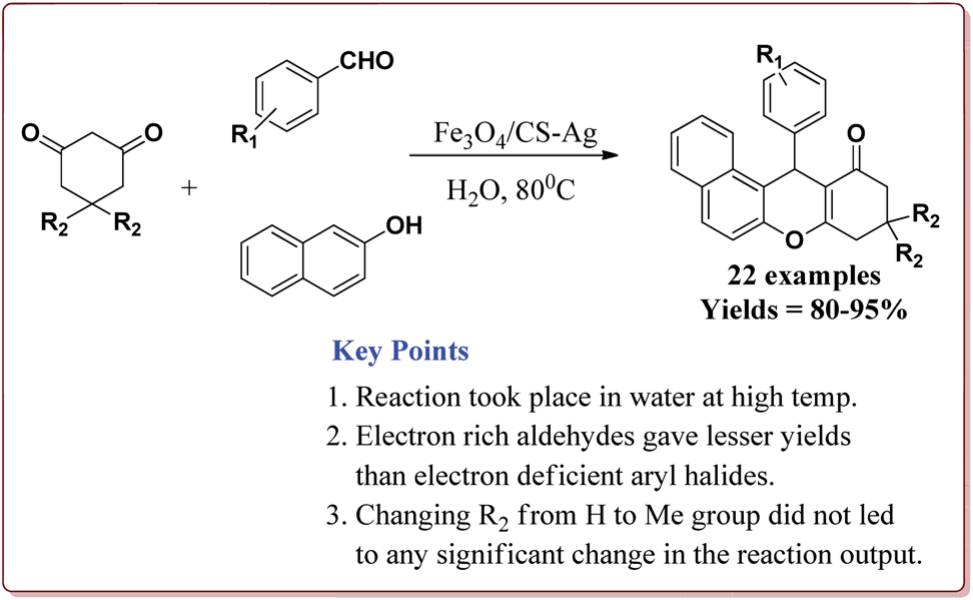
Fe_3_O_4_/CS-Ag catalyzed synthesis of tetrahydrobenzo[α]xanthene-11-ones.

15 g of the prepared catalyst was sufficient for the reaction and could be reused for 7 cycles without any significant loss of its catalytic activity. Further, the reactions proceeded in water at high temperature where electron deficient aryl aldehyde derivatives gave better results (Scheme 47).^198^

**Scheme 48:**
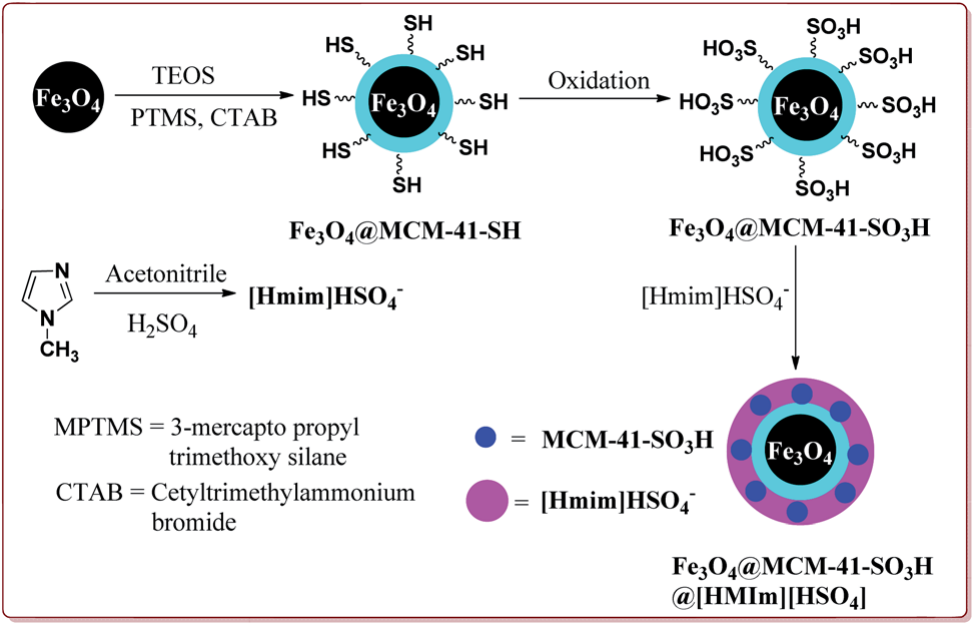
Preparation of Fe_3_O_4_@MCM-41-SO_3_H@[HMIm][HSO_4_] nanocatalyst.

Kefayati *et al.* have reported the synthesis of Fe_3_O_4_@MCM-41-SO_3_H@[HMIm][HSO_4_] as a magnetically separable catalyst for the preparation of spiro[benzochromeno[2,3-*d*]pyrimidin-indolines]. The catalyst was prepared by reacting Fe_3_O_4_@MCM-41-SO_3_H and [Hmim][HSO_4_] at room temperature for 5 hours. Both the reactants were prepared by already reported methods (Scheme 48).^199–201^

**Scheme 49:**
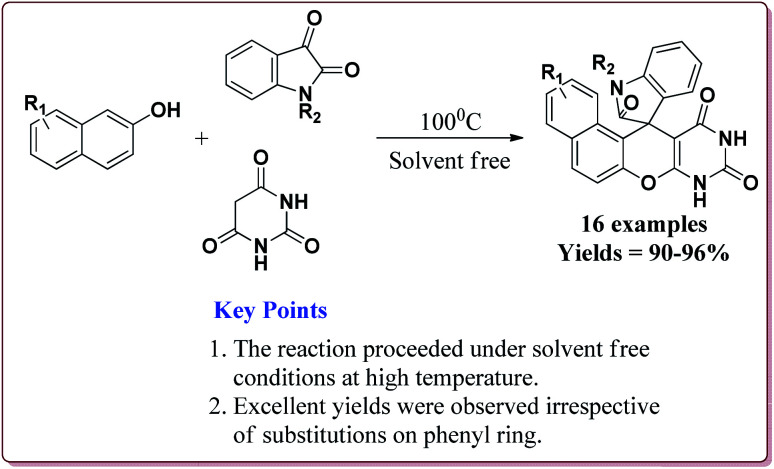
Preparation of spiro[benzochromeno[2,3-*d*]pyrimidin-indolines] catalyzed by Fe_3_O_4_@MCM-41-SO_3_H@[HMIm][HSO_4_].

80 mg of the catalyst was able to catalyse the reactions under solvent free conditions at higher temperatures. The reactions gave excellent results irrespective of the substituents on the aromatic ring. Further, the catalyst could be reused for 5 cycles without loss of its catalytic activity (Scheme 49).^202^

**Scheme 50:**
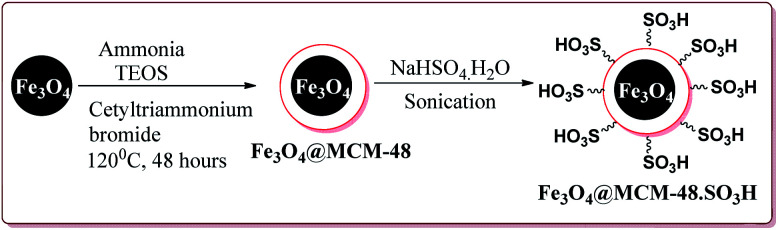
Preparation of Fe_3_O_4_@MCM-48–SO_3_H nanocatalyst.

In an another publication, Kefayati *et al.* have reported the use of Fe_3_O_4_@MCM-48–SO_3_H nanoparticles as a catalyst for the synthesis of benzo[*f*]chromeno[2,3-*d*]pyrimidinones. The catalyst was prepared by the reaction of Fe_3_O_4_ nanoparticles with TEOS and cetyltriammonium bromide to give Fe_3_O_4_@MCM-48 which was then reacted with NaHSO_4_·H_2_O under sonication to afford Fe_3_O_4_@MCM-48–SO_3_H (Scheme 50).

**Scheme 51:**
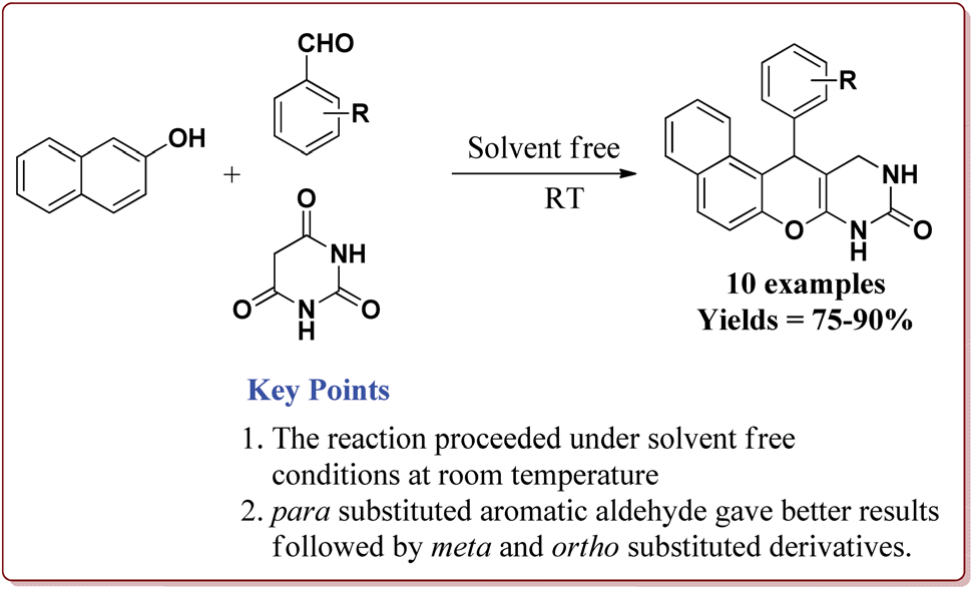
Preparation of benzo[*f*]chromeno[2,3-*d*]pyrimidinones catalyzed by Fe_3_O_4_@MCM-48–SO_3_H nanocatalyst.

The catalyst could catalyse the synthesis of benzo[*f*]chromeno[2,3-*d*]pyrimidinones at room temperature using solvent free conditions where *para* substituted aromatic aldehyde derivatives gave better yields followed by *meta* and *ortho* substituted derivatives. Further, the only 50 mg of the catalyst was required to catalyze the reaction and could be used for 5 cycles without any loss in its activity (Scheme 51).^203^

## 5. Concluding remarks and outlook

As discussed in this review, a significant amount of research work has been done in the field of magnetically recoverable catalysts for carrying out various organic transformations. These catalysts offer many advantages such as excellent reaction output, stability, easily separability and reusability with minimal loss of activity. In addition, silica coated nanocatalysts offer the use of wide range of functional groups. However, there are number of reactions where the reaction mechanisms are still unknown and therefore more studies in this area could lead to explain any unexpected results which have been reported in this filed. In addition, there is a need to develop the catalysts which give required stereoselective products. Also, catalyst leaching is one of the important aspects that lead to decrease in the catalytic activity of the catalyst on repeated use in some cases and this area need much attention for future studies and therefore, this field will remain the focus of the research in the upcoming years also.

## Funding information

This review does not receive any specific grant from funding agencies in the public commercial.

## Conflicts of interest

There are no conflicts to declare.

## Supplementary Material
